# Activation of Dun1 in response to nuclear DNA instability accounts for the increase in mitochondrial point mutations in Rad27/FEN1 deficient *S*. *cerevisiae*

**DOI:** 10.1371/journal.pone.0180153

**Published:** 2017-07-05

**Authors:** Aneta Kaniak-Golik, Renata Kuberska, Piotr Dzierzbicki, Ewa Sledziewska-Gojska

**Affiliations:** Institute of Biochemistry and Biophysics Polish Academy of Sciences, Warsaw, Poland; University of Alabama at Birmingham, UNITED STATES

## Abstract

Rad27/FEN1 nuclease that plays important roles in the maintenance of DNA stability in the nucleus has recently been shown to reside in mitochondria. Accordingly, it has been established that Rad27 deficiency causes increased mutagenesis, but decreased microsatellite instability and homologous recombination in mitochondria. Our current analysis of mutations leading to erythromycin resistance indicates that only some of them arise in mitochondrial DNA and that the GC→AT transition is a hallmark of the mitochondrial mutagenesis in *rad27* null background. We also show that the mitochondrial mutator phenotype resulting from Rad27 deficiency entirely depends on the DNA damage checkpoint kinase Dun1. *DUN1* inactivation suppresses the mitochondrial mutator phenotype caused by Rad27 deficiency and this suppression is eliminated at least in part by subsequent deletion of *SML1* encoding a repressor of ribonucleotide reductase. We conclude that Rad27 deficiency causes a mitochondrial mutator phenotype *via* activation of DNA damage checkpoint kinase Dun1 and that a Dun1-mediated increase of dNTP pools contributes to this phenomenon. These results point to the nuclear DNA instability as the source of mitochondrial mutagenesis. Consistently, we show that mitochondrial mutations occurring more frequently in yeast devoid of Rrm3, a DNA helicase involved in rDNA replication, are also dependent on Dun1. In addition, we have established that overproduction of Exo1, which suppresses DNA damage sensitivity and replication stress in nuclei of Rad27 deficient cells, but does not enter mitochondria, suppresses the mitochondrial mutagenesis. Exo1 overproduction restores also a great part of allelic recombination and microsatellite instability in mitochondria of Rad27 deficient cells. In contrast, the overproduction of Exo1 does not influence mitochondrial direct-repeat mediated deletions in *rad27* null background, pointing to this homologous recombination pathway as the direct target of Rad27 activity in mitochondria.

## Introduction

In eukaryotic cells, mitochondria are essential organelles that provide energy in the form of ATP and supply various biosynthetic intermediates and co-factors necessary for cellular metabolism. Mitochondria retain their own stripped-down genomes that are maintained in the majority of eukaryotic cells by factors encoded entirely by the nuclear genome. The central function of mitochondrial DNA (mtDNA) is the production of a few proteins that are necessary for respiration, in particular for ATP synthesis by mitochondria through oxidative phosphorylation. Respiration, in turn, contributes to the essential function of mitochondria, because the electron transport chain activity coupled to oxidative phosphorylation is one of the main pathways for generation of a mitochondrial transmembrane potential ΔΨ that is needed for import of the essential proteins to mitochondria and for mitochondrial biogenesis [1, 2]. Though cells of yeast *Saccharomyces cerevisiae* can survive without respiration and mtDNA is dispensable for their viability in laboratory conditions, loss of mtDNA in the budding yeast leads to nuclear genome instability through a defect of essential iron-sulfur cluster biogenesis that is dependent on normal mitochondrial function [3]. In addition, properly respiring mitochondria are required during chronological aging [4]. Thus, the maintenance of functional mtDNA is crucial for long-term survival and homeostasis of eukaryotic cells.

The stability of the mitochondrial genome relies on the efficient functioning of various mitochondrial pathways that recognize, remove and repair damage in mtDNA [5]. If not repaired, lesions in mtDNA may result in mutations, which in humans may cause a variety of hereditary diseases such as mitochondrial encephalomyopathies and neuropathies, and are associated with the pathogenesis of a variety of complex disorders including heart disease, neurodegenerative diseases such as Parkinson’s, Alzheimer’s and Huntington’s, and other neurological disorders [6, 7]. The accumulation of somatic mutations in mtDNA is also characteristic of both normal ageing process and cancer [8, 9]. Interestingly, in the wake of recent studies on mtDNA mutagenesis, it has become increasingly evident that, contrary to the long-held proposition, the main source of somatic mtDNA mutations is not oxidative damage to mtDNA, but rather replication errors or spontaneous base hydrolysis (reviewed in [10]). This paradigm shift in the research on mtDNA stability underscores the importance of studying mitochondrial pathways that monitor the process of mtDNA replication and those that repair the spontaneous base decay in mtDNA.

Replication and repair of mtDNA require the involvement of nuclease activities to process different mtDNA intermediate structures and to maintain the stability of the mitochondrial genome. One of the nucleases that has been implicated in mtDNA repair is Rad27. The Rad27 nuclease is well characterized as an enzyme, but its roles *in vivo* in maintaining the stability of mtDNA are poorly understood. Rad27, the *S*. *cerevisiae* ortholog of mammalian FEN1, has three nucleolytic activities: 5’-endonuclease, 5’-exonuclease and gap endonuclease (reviewed in [11, 12]). It is involved in many nuclear DNA transactions including cleavage of 5’-DNA flaps created during Okazaki fragment processing, processing of intermediates during long-patch base excision repair (LP-BER), prevention of sequence duplications and repeat sequence expansions, as well as in DSB repair [13–15]. Accordingly, deficiency of Rad27/Fen1 causes genetic instability (ref. as above) and strong induction of checkpoint signaling pathway [16, 17].

It has been reported that Rad27 (FEN1) functions in mitochondria of both yeast and mammalian cells [18–20]. However, the involvement of FEN1 in mitochondrial LP-BER is a matter of controversy. On the one hand, Liu and colleagues using *in vitro* assays have shown that the LP-BER activity of mammalian mitochondrial extracts was strongly diminished upon the removal of FEN1 [19]. On the other hand, it was reported that the LP-BER activity was only marginally affected in FEN1-depleted mitochondrial extracts, suggesting the involvement of another 5’-exo/endonuclease in mitochondrial LP-BER [21]. Moreover, mitochondrial BER of uracil and AP sites was demonstrated by Akbari and collaborators to occur by both single-nucleotide insertion and long-patch repair DNA synthesis [18]. However, these authors also showed that although mitochondrial extracts were able to remove 5' protruding DNA flaps formed during LP-BER by a "flap endonuclease like" activity, FEN1 was not present in mitochondria. In addition, EXOG, a 5’-exo/endonuclease unique to the mitochondria has been implicated in the removal of the 5’-blocking residue, and was shown to be required for repairing endogenous SSBs in the mitochondrial genome [22]. The latter report demonstrated that FEN1 depletion did not affect the integrity of the mitochondrial genome. The consequences of FEN1 depletion on the mitochondrial point mutagenesis in mammalian cells were not investigated.

*S*. *cerevisiae rad27* null mutants display an increased rate of base substitutions in mtDNA, but at the same time exhibit a decrease in mitochondrial direct-repeat mediated deletions (DRMD) and a decrease in mitochondrial di-nucleotide repeat instability [20]. The two latter mitochondrial phenotypes of *rad27*Δ mutants are in stark contrast to their nuclear phenotypes, since inactivation of *RAD27* leads to hyper-recombination of the nuclear genome [23] and elevated rates of nuclear di- and trinucleotide tract instability [24, 25]. The mechanisms underlying the mitochondrial phenotypes in *rad27*Δ strains are not understood. Our current study points to the recombination mechanism responsible for DRMD as the main direct target of mitochondrial activity of Rad27. In contrast, we show that the increase in mitochondrial point mutagenesis in Rad27 deficient cells is a consequence of nuclear DNA instability, underscoring the crosstalk between the nucleus and mitochondria *via* Dun1-dependent activities.

## Materials and methods

### Strains, plasmids and growth conditions

All strains that were used in this study are listed in Table 1. To estimate the stability of mitochondrial microsatellite repeat sequences, we used a strain harbouring in its mtDNA the *arg8*^*m*^::(GT)_16_(+2) reporter in place of the *COX3* gene [26]. The strain (YAK136) was obtained by cytoduction of mtDNA from the original strain with the mitochondrial reporter (strain CAB193-1; reference as above) into a *rho*^0^ *MAT***a** *arg8*Δ::*URA3* strain derived from our wild-type strain FF18733 *via* a *MAT***α** *kar1-1* strain (DFS160 [27]). The *URA3* cassette for inactivation of the nuclear *ARG8* gene was amplified by a PCR on the template of a genomic DNA extracted from the DFS160 strain (primers ARG8_A and OA54 in S1 Table). For assays of mitochondrial allelic homologous recombination, we used the method described in [28] with a parental pair of wild-type strains: YAK349 and YAK243. YAK243 was obtained in a way analogous to that described above for the construction of YAK136, i.e. through cytoduction of mtDNA from CAB183-1 (with the *arg8*^*m*^::(AT)_16_(+1) reporter replacing the *COX3* gene) into a *rho*^0^ *MAT***a** *arg8*Δ::*URA3* strain derived from FF18733 *via* a DFS160 carrier strain. YAK349, in turn, was isolated through cytoduction of mtDNA from YAK29/1 (via a *MAT***a** *kar1-1* strain, JC8/55) into a *rho*^0^ *MAT***α** *arg8*Δ::*URA3* strain derived from PJD1 [29]. The procedure used for those and other cytoduction experiments is described in Supplemental Information (S1 Text).

**Table 1:** *S*. *cerevisiae* strains used in this study.

*Strain*	*Relevant nuclear genotype*^a^/ *mitochondrial genotype*	*Source*
FF18733	*MAT***a** *leu2-3*,*112 trp1-289 ura3-52 his7 lys1-1/ ρ*^*+*^	[30]
FF18734	*MATα leu2-3*,*112 trp1-289 ura3-52 his7 lys1-1/ ρ*^*+*^	[30]
DFS160	*MATα ade2-101 leu2 ura3-52 arg8*Δ::*URA3 kar1-1/ ρ*^*0*^	[27]
JC8/55	*MAT***a** *leu1 kar-1-1/ ρ*^*0*^	[31, 32]
PJD1	*MATα ade2-101 ade3-24 leu2-3*, *112 ura3-52/ ρ*^*+*^	[29]
CAB193-1	*MAT***a** *leu2-3*,*112 ura3-52 lys2 his7 arg8*::*hisG/ ρ*^*+*^ *cox3*::*arg8*^*m*^::*(GT)*_*16*_ *(+2)*	[26]
CAB183-1	*MAT***a** *leu2-3*,*112 ura3-52 lys2 his7 arg8*::*hisG/ ρ*^*+*^ *cox3*::*arg8*^*m*^::*(AT)*_*16*_ *(+1)*	[26]
TF236	*MAT***a** *ino1*::*HIS3 arg8*::*hisG pet9 ura3-52 lys2/ ρ*^*+*^ *cox3*::*arg8m-1*	[33]
GW22	*MAT***a** *lys2/ ρ*^*+*^ *cox3-421*	[27]
MCC259	*MATα ade2-101 ura3-52 kar1-1/ ρ¯*^*¯*^ *[COX3]*	[26]
EAS748	*MAT***a** *ura3-52 leu2-3*,*112 lys2 his3 arg8*::*hisG/ ρ*^*+*^ Rep96::*ARG8*^*m*^::*cox2*	[34]
YAK254^g^	FF18733 *rad27*Δ::*kanMX4/ ρ*^*+*^	[35]
YAK1070	*MATα leu2-3*,*112 trp1-289 ura3-52 HIS7*^*+*^ *lys1-1/ ρ*^*0*^	This study
YAK225	*MAT***a/** *MATα leu2-3*,*112/leu2-3*,*112 trp1-289/trp1-289 ura3-52/ura3-52 his7/his7 lys1-1*/*lys1-1/ ρ*^*+*^	This study
YAK385	YAK225 *rad27*Δ::*kanMX4/ RAD27*^*+*^*/ ρ*^*+*^	This study
YAK1337 ^a^	*rrm3*Δ::*hphMX4/ ρ*^*+*^	This study
YAK1339 ^a^	*msh1-R813W rrm3*Δ::*hphMX4/ ρ*^*+*^	This study
YAK1405	FF18733 *rad27*Δ::*hphMX4/ ρ*^*+*^	This study
YAK1441^a^	*rad27*Δ::*hphMX4/ ρ*^*+*^	This study
YAK1446 ^a^	*din7*Δ::*natMX4/ ρ*^*+*^	This study
YAK1444 ^a^	*rad27*Δ::*hphMX4 din7*Δ::*natMX4/ ρ*^*+*^	This study
YAK1445 ^a^	*msh1-R813W/ ρ*^*+*^	This study
YAK1671 ^a^	*dun1*Δ::*kanMX4/ ρ*^*+*^	This study
YAK1682 ^a^	*dun1*Δ::*kanMX4 rad27*Δ::*hphMX4/ ρ*^*+*^	This study
YAK1696 ^a^	*dun1*Δ::*kanMX4 sml1*Δ::*natMX4/ ρ*^*+*^	This study
YAK1752 ^a^	*dun1*Δ::*kanMX4 sml1*Δ::*natMX4 rad27*Δ::*hphMX4/ ρ*^*+*^	This study
YAK1884 ^a^	*msh1-R813W rrm3*Δ::*hphMX4 dun1*Δ::*kanMX4/ ρ*^*+*^	This study
YAK136	FF18733 *arg8*Δ::*URA3/ ρ*^*+*^ *cox3*::*arg8*^*m*^::*(GT)*_*16*_ *(+2)*	This study
YAK1629	FF18733 *arg8*Δ::*URA3 rad27*Δ::*hphMX4/ ρ*^*+*^ *cox3*::*arg8*^*m*^::*(GT)*_*16*_ *(+2)*	This study
YAK349	PJD1 *arg8*Δ::*URA3/ ρ*^*+*^ *cox3*::*arg8m-1*	This study
YAK243	FF18733 *arg8*Δ::*URA3/ ρ*^*+*^ *cox3*::*arg8*^*m*^::*(AT)*_*16*_ *(+1)*	This study
YAK1094	PJD1 *arg8*Δ::*URA3 rad27*Δ::*kanMX6/ ρ*^*+*^ *cox3*::*arg8*^*m*^*-1*	This study
YAK1097	FF18733 *arg8Δ*::*URA3 rad27*Δ::*kanMX6/ ρ*^*+*^ *cox3*::*arg8*^*m*^::*(AT)*_*16*_ *(+1)*	This study
YAK1659	EAS748 *rad27*Δ::*kanMX6/ ρ*^*+*^ Rep96::*ARG8*^*m*^::*cox2*	This study

^a^ strains isolated after dissection of tetrads produced during sporulation of several heterozygous strains; mating type and auxotrophic markers as in the FF18733 strain.

Erythromycin-resistance (E^R^) mutagenesis was tested in the genetic context of the FF18733 strain [30]. The strain YAK225 was isolated by crossing FF18733 with the isogenic FF18734 strain [30]. Strains that are indicated in Table 1 as segregants were isolated after dissecting tetrads issued from sporulation of heterozygous strains that had been obtained by crossing appropriate haploid strains or transformation of diploid strains (congenic with FF18733 and FF18734) with deletion cassettes. All genetic manipulations were done essentially as described by Amberg et al. [36]. To introduce *kanMX4-*, *hphMX4*- or *natMX4*-marked deletions into our strains, we used mainly the PCR-mediated strategy described earlier [35] with antibiotic-resistance cassettes according to [37] and ORF-specific and marker-specific primers as listed in S1 Table. Deletion cassettes to inactivate studied genes with the *kanMX4* maker gene were derived from strains in the Institute collection of yeast deletions from the Saccharomyces Genome Deletion Project [38], cited earlier in [39]. For inactivations of the *RAD27* gene, we followed either the above-mentioned procedure, using cassettes with long flanks (primers RAD27_A and RAD27_D; S1 Table) or, as a cautionary measure to avoid introducing inadvertent modifications in the promoter of the adjacent *APN1* gene, PCR-amplified *kanMX6* or *hphMX4* cassettes flanked with shorter flanks (40 bp long). For *de novo* amplification of the *rad27*Δ::*kanMX6* cassette with short flanks, using the Phusion polymerase (Thermo Scientific), we utilized primers OA117 and OA118 (S1 Table) and plasmid pFA6a-kanMX [40] as a template. For amplification of the *rad27*Δ::*hphMX4* cassette with short flanks, we used primers OA136 and OA137 (S1 Table) and, as a template, genomic DNA extracted from hygromycin B-resistant segregants obtained in tetrad dissection of the YAK385 strain (heterozygous *rad27*Δ::*kanMX4*; Table 1) in which the *kanMX4* marker had been replaced for *hphMX4* according to [37]. *DUN1* deletion was performed by transformation of diploid strains with the *dun1*Δ::*kanMX4* fragment (short flanks) amplified with primers OA152 and OA153 on the template of a genomic DNA extract from the BY4741 *dun1*Δ strain [38]. *SML1* deletion strains (*sml1*Δ::*natMX*) were constructed according to [41], using primers listed in S1 Table. *RRM3* was deleted using an *hphMX4*-marked cassette with long flanks amplified with primers RRM3_A and RRM3_D (S1 Table). All isolates were verified by PCR amplifications of modified loci, using primers listed in S1 Table, following the methodology described in Johnston et al. [42].

The wild-type copy of the *MSH1*^+^ gene was replaced by the *msh1-R813W* allele using the two-step gene replacement method in which *Eco*RI-linearized plasmid pCK14 [28] was integrated into the chromosomal *MSH1* locus in the FF18733 strain (Table 1). The excision of the plasmid was selected in the resulting transformants by passaging twice on a medium with 5-fluoroorotic acid (5-FOA) [36]. Since the R813W substitution in the *MSH1*-encoded protein had been designed to create a *Pvu*II site in the mutated allele [43], the presence of the mutant copy of the gene in isolated 5-FOA-resistant strains was verified by *Pvu*II digestion of PCR products amplified with primers MSH1_C1 and MSH1_D (S1 Table) on genomic DNA isolated from the strains. The verified *msh1-R813W* isolates were crossed with FF18734 to obtain heterozygous diploid strains. The *Pvu*II digestion of MSH1_C1-MSH1_D products was also used for tracking *msh1-R813W* allele segregations in meiotic progeny obtained by sporulation of *msh1-R813W* heterozygous strains.

To obtain centromeric plasmid pCK62 encoding *RAD27*, the *Bgl*II fragment containing the gene and the *LEU2* marker was isolated from pEAI141 [13], kindly provided by Eric Alani, and inserted into pFL38 [44] to substitute for the *URA3* containing–*Bgl*II fragment. The resulting plasmid was named pCK54. Subsequently, the *Bam*HI site derived from the original multiple cloning site of pFL38 was removed by double cleavage with *Sma*I and *Xba*I, followed by Klenow treatment and re-ligation. A plasmid isolated after the *Bam*HI site removal was named pCK62. To convert the wild-type allele of *RAD27* into to the rad27-R325* allele, pCK62 was mutagenized, following *Bam*HI cleavage and dephosphorylation with shrimp alkaline phosphatase (Thermo Scientific), by a gap repair recombination using a pair of overlapping mutagenic PCR-generated fragments. One mutagenic fragment was amplified with a pair of primers RAD27_C (S1 Table) and OA112 (ditto) on the template of pCK54 plasmid using the Phusion polymerase (Thermo Scientific). The other fragment was also generated in a Phusion-catalyzed PCR with the pair of primers OA111 (S1 Table) and RAD27_D (ditto) on the template of pCK54. The final construct with the rad27-R325* allele was verified by sequencing and named pCK64.

To obtain a plasmid encoding the fusion of α lacZ fragment with the C-terminus of Exo1, we based our construction on the plasmid p680 (a generous gift from Ophry Pines). The plasmid contains a gene encoding a fusion protein Aco1-α [45] cloned in pRS425-GAL1 [46]. For the construction, p680 was linearized with *Pst*I and dephosphorylated. Subsequently, the cut plasmid was gap-repaired by recombination with a PCR Expand High-Fidelity Taq polymerase (Roche)-amplified fragment, containing *EXO1* open-reading frame (ORF), obtained with primers OA85 and OA86 (S1 Table) on the template of a genomic DNA extract from the wild-type strain FF18733. The fragment for the gap repair of the linearized p680 contained the sequence of *EXO1* ORF, without its STOP codon, embedded in appropriate sequences homologous to p680 that were directing the recombination. 5’-terminus of *EXO1* ORF sequence was linked to the *GAL1* promoter. The 3’-terminal end of the gene (omitting the *EXO1* STOP codon) was joined to the sequence coding for the α *lacZ* fragment (with a linker peptide of 4 amino acids, Pro-Gly-Gln-Thr, as described in [47]). Thus, in this gap repair construction, *EXO1* ORF (without its STOP codon) replaces the *ACO1* ORF in p680. The final construct pRS425-GAL1-EXO1-α was named pCK48. In an analogous way, we constructed pRS425-GAL1-RAD51-α plasmid (pCK40), using primers OA68 and OA69 (S1 Table).

To construct pYES2-CTA1, which enables the expression of the *CTA1* gene under its native promoter from a *URA3*-containing 2μ plasmid, the 2.8 kb-*Eco*RI fragment, containing the gene, was recloned from YEp352-ura3Δ::kanMX-CTA1 [35] into pYES2 linearized with the same restriction enzyme. The final plasmid pYES2-CTA1 was named pCK78.

Plasmids: pYES-DEST52-EXO1-V5-6xHis and pYES-DEST52-RAD27-V5-6xHis were kindly provided by Miguel Blanco and Stephen West [48]. Plasmid pRDK480 (*EXO1* on 2μ) was a generous gift from Richard Kolodner [49].

Media for growth (YPD, YPG as well as synthetic complete media for plasmid selection) and sporulation are essentially as described [36]. For synthetic complete media with appropriate requirement omissions, supplement drop-out mixes were used from BioShop (distributed by Lab Empire). For experiments with strains harbouring the *ARG8*^*m*^ gene, we used a drop-out supplement mix according to [50]. For preparing synthetic media without uracil, a formula based on yeast nitrogen base with ammonium sulfate (Difco) and supplemented with casamino acids at 0.5% and tryptophan (as below) was also used. For nuclear mutagenesis assays to measure frequencies of forward mutations in the *CAN1* gene eliciting resistance to canavanine, cultures of tested strains were plated on a minimal glucose medium with all the auxotrophic requirements, unless a particular requirement (uracil or leucine) was omitted in case of plasmid selection, and supplemented with canavanine at 60 mg/l. Auxotrophic requirements were added to synthetic media at concentrations recommended in [51]: tryptophan at 80 mg/l, leucine at 217 mg/l, uracil at 22.4 mg/l, histidine at 47 mg/l, lysine at 162 mg/l. Non-respiring *petite* colonies were scored on YPG medium supplemented with 0.1% glucose (YPGd). Erythromycin-resistant (E^R^) mutants were scored on YPG medium (buffered at pH 6.2) containing 4 g/l erythromycin. For plasmid amplification in bacteria, *Escherichia coli* strain XL1Blue (Stratagene) was used.

### Isolation and genetic analysis of erythromycin-resistant mutants for sequencing analysis

For this experiment, cells were cultured by a method proposed in [52] with some modifications. Cells were spread on YPG plates without sub-cloning directly from frozen stocks at -80°C and incubated for 4 days at 28°C. Subsequently, cells were re-plated on YPD for single colonies. After 3-day incubation of the plates at 28°C, individual colonies were suspended in 20 μl sterile water. A 5-μl-aliquot of the suspension was diluted appropriately in water and plated on YPGd to assess, upon incubation for several days at 28°C, the number of *rho*^+^ and *petite* cells in tested suspensions (using the tetrazolium overlay method [53]). The rest of a cell suspension was patched on YPG+erythromycin plates. Cell patches were arrayed in a 12-node grid per plate, i.e. one-colony suspension was patched on a 2-cm^2^ square in the grid and 12 independent gridded YPD-grown colonies per strain were tested in parallel for resistance to erythromycin on one 8.5 cm-diameter plate. Altogether, 12 such plates for the wild-type strain (FF18733) with 12 independent 1-colony-patches were processed and 10 plates for *rad27*Δ (YAK254; Table 1). Plates were incubated at 28°C and scored for erythromycin-resistant colonies after 7 days and 14 days of incubation. Mean values of E^R^ mutant frequencies are reported for this experiment in Table 2. 95% confidence intervals of the mean (CI 95%) were calculated, using the GraphPad Prism 6 software. To determine the localization of E^R^ mutations and ensure the independent origin of analyzed clones, for all the patches, only one E^R^ colony per patch was picked up and purified by an additional passage on fresh YPG+erythromycin plates for further analyses. Each isolated mutant was subjected to a cytoduction test (S1 Text). In addition, to isolate heterozygous strains for tracking the E^R^ trait in meiotic progeny, E^R^ isolates lacking the *RAD27* gene were also crossed in parallel with a *rho*^0^ derivative of the wild-type strain FF18733 carrying a prototrophic allele of the *HIS7* gene (YAK1070; Table 1). Diploid cells (His^+^ G418^R^) generated in the crosses were selected as described in S1 Text. Tetrads issued from sporulation of isolated diploid clones were dissected on YPD plates and spore clones originating from complete tetrads were analyzed for segregating markers.

**Table 2:** The effect of Rad27 deficiency on the frequencies of total and mitochondrial erythromycin-resistant (E^R^) mutants.

Strain	E^R^/10^7^ *rho*^+^				E^R^/10^7^ *rho*^+^			
	after 7 days				after 14 days			
	Total	(CI 95%)	Mitochondrial	(fold)	Total	(CI 95%)	Mitochondrial	(fold)
Wild-type	2.01	(1.24–2.78)	2.01	(1)	3.82	(2.78–4.86)	3.82	(1)
*rad27*Δ	5.07	(2.78–7.36)	5.07	(2.5)	60.95	(37.21–84.68)	31.05	(8.1)

Colonies of FF18733 (wt) and YAK254 (*rad27*Δ) cells were grown on YPD medium as described in Materials and methods and re-plated in gridded patches on YPG medium with erythromycin (WT: n = 144; *rad27*Δ: n = 120; n: number of colonies analyzed as patches on the erythromycin-containing plates). Mean values of frequencies of total E^R^ mutants were determined after 7 days of incubation and a week later. 95% confidence intervals of the mean (CI 95%) is shown in parentheses. In the columns titled “Mitochondrial”, in parentheses: fold increase of a mitochondrial E^R^ mutation frequency in comparison to that of the wild-type strain. Independent E^R^ mutants (only 1 per patch) were picked up at indicated incubation times and tested by a cytoduction test. The frequencies of mitochondrial mutants after 7 days of incubation for each strain were calculated from the total frequencies after 7 days corrected by the percentage of E^R^ mutations that could be transmitted by cytoduction as determined in the cytoduction test. To calculate the frequency of final mitochondrial mutants after 14 days, the frequency of mitochondrial mutants appearing between the 7^th^ day and the 14^th^ day of incubation (late mitochondrial mutants), calculated from the difference of total frequencies determined after 14 days and after 7 days, and corrected by the percentage of E^R^ mutations that could be transmitted by cytoduction tests, was added to the frequencies of mitochondrial E^R^ after 7 days (early mitochondrial mutants).

### Sequencing of mutations in the 21S rRNA locus in mtDNA of mitochondrial erythromycin resistant mutants

Total genomic DNA extracts were prepared from strains with E^R^ mutations localized in mitochondria by the cytoduction test. For extractions (from 3 ml of YPD culture of strain per extraction), a scaled-down modification of the method with a zymolyase digestion (a yeast DNA miniprep) described in [36] proved to give DNA extracts of a sufficient yield and quality for subsequent PCR amplifications preceding the final sequencing step. The primer pair ERY-1 and ERY-2, according to [54], was used for PCR amplification of the mitochondrial 21S rRNA and sequencing. The primers amplify a mtDNA fragment from the nucleotide at position 1731 to the nucleotide at position 2293 (the coordinates correspond to the 21S rRNA sequence from Saccharomyces Genome Database [55] and not to the GeneBank sequence cited in [54]). Before sequencing, PCR-amplified fragments were purified on Clean Up (A&A Biotechnology) columns. Reported sequence changes in the 21S rRNA were confirmed by two sequencing reactions with both primers.

### Determination of erythromycin-resistant (E^R^) mutant frequencies

The frequency of E^R^ mutants was estimated as described in the subsection “**Isolation and genetic analysis of erythromycin-resistant mutants for sequencing analysis”** (results in Table 2) or in liquid cultures. To start cultures for mutagenesis assays, 3–4 day old colonies growing on YPG plates were inoculated independently into 3 ml of an indicated medium. In experiments with strains harbouring plasmids, colonies with plasmid-transformed cells were picked up directly from transformation plates to inoculate cultures in appropriate selective media. For experiments with plasmids coding for genes under the *GAL1* promoter, transformants were isolated on plates with a complete synthetic selective medium with 2% sucrose as the carbon source. Cells from late logarithmic cultures (grown on glucose-containing media for 16 hours, or on media with galactose as the carbon source for 24 hours) were harvested by centrifugation, washed with sterile water and plated on YPG+erythromycin medium. Appropriate dilutions of the cultures were also spread on YPGd plates to calculate the number of *petite* and *grande* (*rho*^+^) cells. All plates were incubated at 28°C. Colonies on YPGd plates were counted after 3–5 days of incubation and scored as *rho*^+^ or *rho*¯/*rho*^0^ (i.e. *petite*) by the tetrazolium overlay method [53]. E^R^ colonies were scored after indicated incubation times. Median values derived from several combined experiments with at least 10 independent cultures of each strain were used to determine the frequency of mutants resistant to erythromycin calculated per number of *rho*^+^ cells. P values for statistical significance of any differences in mutant frequencies between pairs of strains were determined by calculating the nonparametric Mann–Whitney statistics, using the GraphPad Prism 6 program. The same software was used for drawing the graphs and calculating 95% confidence intervals of median values.

We also used the same statistical analysis for processing results of other mutagenesis assays in the current study, with the exception of mitochondrial allelic recombination (details in the section **Mitochondrial allelic recombination tests**) and the erythromycin-resistance mutagenesis control test (results in Table 2) accompanying the mtDNA sequencing analysis, as described in the preceding section **Isolation and genetic analysis of erythromycin-resistant mutants for sequencing analysis.**

### Determination of canavanine-resistant (Can^R^) mutant frequencies

To measure levels of nuclear mutagenesis in tested strains, mutations in the *CAN1* gene conferring resistance to canavanine were selected on a medium with canavanine, as described in the subsection **Strains, plasmids and growth conditions.** Cells were grown in rich or selective media (as indicated), harvested, rinsed with water and plated (around 2.5 x 10^7^ cells/5.5 cm diameter-plate). Can^R^ mutants were scored after 5 days of incubation at 28°C. Median frequency values were analyzed as described above.

### Determination of phenotypes in strains harbouring variants of the mitochondrial reporter gene *ARG8*^m^

#### Microsatellite stability assays

Tests of the stability of GT dinucleotide repeats inserted out of frame in the mitochondrial reporter gene *ARG8*^m^ were performed in the YAK136 strain and its derivatives (Table 1). Strains transformed with plasmids used in this study were grown overnight in a synthetic complete glucose medium without leucine. Cells were diluted appropriately to determine culture densities by plating the dilutions on the respective selective medium. For the proper test to determine the number of Arg^+^ prototrophs in cultures, cells from appropriate culture aliquots (100 to 200 μl of a culture) were harvested by centrifugation, rinsed with water and plated on double omission plates with a synthetic complete dextrose medium without leucine and arginine. Plates were incubated at 28°C and colonies growing on plates were scored on the 3^rd^ day of incubation. Median Arg^+^ frequency values were analyzed as described above.

#### Mitochondrial allelic recombination tests

To estimate levels of allelic mitochondrial recombination, we performed crosses as described in [28] with two major modifications: 1) the use of parental strains described in the 1^st^ subsection of Materials and Methods; 2) altered conditions of parental pre-cultures for crosses. Since in preliminary experiments *rho*^+^ mtDNA turned out to be highly unstable in transformed cells of MAT**a** strain YAK243 *rad27*Δ (YAK1097; Table 1) growing in a medium selective for the presence of tested plasmids (synthetic complete without leucine with glucose as the carbon source), and, in contrast, both MATα strains, WT and its *rad27*Δ, retained their *rho*^+^ mtDNA acceptably well in these conditions, we modified the original procedure for crosses by growing precultures of untransformed MAT**a** parental strains in rich glucose medium (YPD) and precultures of transformed MATα parental strains in the selective medium. After the crosses, resulting diploid cells were selected in a minimal glucose-containing medium supplemented only with arginine, thus in conditions selective for the presence of the plasmids derived only from MATα parental cells. As described in [28], in parallel to those crosses, all parental cultures were also analyzed by crosses with tester strains, GW22 and MCC259 (Table 1), to establish the percentage of *rho*^+^ cells for normalization of raw Arg^+^ frequencies in final diploid cultures. Mean values are reported and P values of observed differences were calculated using the unpaired t-test statistics (GraphPad Prism 6).

#### Direct-repeat recombination assays

To establish levels of direct-repeat recombination (DRMD) in mtDNA, we used the DRMD strain EAS748 with the mitochondrial reporter Rep96::*ARG8*^*m*^::*cox2* [34]. For transformation of this strain and its *rad27*Δ derivative (YAK1659; Table 1) with tested plasmids, cells were cultured in a synthetic complete glucose medium without arginine to select for *rho*^+^ mtDNA with the *ARG8*^*m*^ reporter. Transformants were plated on double omission synthetic complete (lacking leucine and arginine) glucose medium. When transformant colonies had grown (usually after 3 days at 28°C), they were pooled and frozen in 15%-glycerol stocks at –80°C. Cells were thawed by spreading a frozen aliquot of cell suspension for single colonies on plates with a synthetic complete single omission (without leucine) glucose medium. Plates were incubated for 3 days (sharp!) at 28°C. Colonies grown on the plates were suspended individually in 100 μl of sterile water. Appropriate aliquots of cell suspensions were withdrawn to make dilutions in water for plating on a synthetic complete glucose medium without leucine (cell density measurement). To select for products of DRMD events, 10–20 μl-aliquots of cell suspensions were plated on synthetic complete glycerol-containing medium without leucine. Plates were incubated for 4 days at 28°C. Colonies growing on them were scored to establish frequencies of respiring cells (arising due to Rep96-mediated deletions of *ARG8*^*m*^) in colonies of thawed transformant strains. Median values of several combined experiments are reported. P values of observed differences were calculated using the Mann-Whitney statistics.

#### Assays of mutagenesis to cycloheximide resistance

To test nuclear mutagenesis in transformant strains harbouring the *ARG8*^m^ reporter gene in their mtDNA, we used a synthetic complete glucose medium (without leucine) with added cycloheximide at the concentration of 1 mg/l for mutant selection. Cycloheximide-resistant colonies were counted after 5 days of incubation 28°C. Median frequencies are reported (S2 Table).

### Colony size estimation

Frozen cells (at –80°C in 15% glicerol) of E^R^ strains were thawed by plating at low density on plates with YPG (pH 6.2) medium with erythromycin and grown for 4 days at 28°C. The plates were imaged using a 24-megapixel (6000 X 4000 pixels) dSLR camera (Sony) with an APS-C sensor (23.5 X 15.6 mm) and a macro lens with 1:1 reproduction ratio (Tamron), resulting in a scale of ~256 pixels/mm. Diameters of colonies (n = 25 for each strain) were measured in pixels using the line tool in the NIH ImageJ2 software, Fiji distribution [56–58]. Colony diameter mean values were compared using the unpaired t-test.

### α-complementation assay

The wild-type haploid strain FF18733 was co-transformed, on the one hand, with plasmids encoding proteins to be tested in a C-terminal fusion with the α fragment of lacZ protein (either pRS425-GAL1-EXO1-α, alias pCK48, or pRS425-GAL1-RAD51-α, alias pCK40) and, on the other hand, with a plasmid encoding either the lacZ ω fragment localized in the cytoplasm (p877, alias pYES/M15 or pWc [59]) or a plasmid coding for the ω fragment directed to mitochondria (p999, alias pWm [59]). For the α-complementation test, transformed strains were cultured in a non-repressing selective synthetic complete medium with 3% glycerol and 0.1% glucose for 2 days. 5 μl-aliquots of undiluted cultures were spotted on a solid selective medium with 2% galactose as a sole carbon source (buffered at pH 7.0) and with X-gal (5-bromo-4-chloro-3-indolyl β-D-galactopyranoside) indicator at 40 mg/l (after the protocol in [36]). The color of yeast colonies, arising due to β-galactosidase activity on the detector plates, was observed after 5 days of incubation at 28°C.

### Fractionation of cells expressing Exo1-6xHIS and analysis of mitochondrial fractions

Cells of a *rad27*Δ::*hphMX* strain (YAK1441) transformed with pYES-DEST52-EXO1-V5-6xHis were grown in selective medium with 2% galactose and 0.1% glucose up to OD_600_ 1.0 and harvested. The mitochondrial fraction was isolated by differential centrifugation according to a described procedure [60]. Samples of the mitochondrial fraction (15 μg/sample) were analyzed by treatment with proteinase K (at concentration 50 μg/ml) in a reaction set-up according to [61]. Digestions were terminated by adding phenylmethane sulfonyl fluoride (PMSF) at 2mM and centrifuged at 12 500 x g for 5 min at 4°C. The pellets were analyzed by 12% SDS-PAGE and a Western blot. His-tagged proteins were detected with the GenScript anti-His monoclonal antibody (at the dilution of 1:5000) and the mitochondrial control Cox2 protein with the monoclonal anti-MTCO2 antibody [4B12A5] from Abcam at the concentration recommended by the manufacturer. As a secondary antibody, a polyclonal goat anti-mouse immunoglobulin with horseradish peroxidase from DakoCytomation (at the dilution of 1:2500) was used, followed by a chemiluminescence detection procedure, as in [28].

## Results

### Verification of mitochondrial phenotypes caused by Rad27 inactivation

*RAD27* inactivation has previously been shown to cause mitochondrial phenotypes, such as: an increase in the frequency of point mutations in mtDNA and a decrease both in mitochondrial recombination and microsatellite instability [20]. To elucidate these phenotypes, we repeated and extended the analysis of the effect of Rad27 deficiency on point mutations in mtDNA leading to the resistance to erythromycin by verification of mitochondrial location of erythromycin-resistant (E^R^) mutations. We also employed systems alternative to those described earlier to measure mitochondrial recombination and microsatellite stability in *rad27*Δ strains.

Mutations conferring resistance to erythromycin are acquired through specific changes in the mitochondrial 21S rRNA gene [62–64] and the acquisition of resistance to this drug is a standard method of measuring point mutagenesis in mtDNA [52, 65]. Table 2 shows that the *rad27*Δ mutant displayed an about 2-fold increase in the frequency of E^R^ mutants as compared to that of the wild-type strain. However, when scoring E^R^ colonies, we observed that incubation of cells on the antibiotic-containing medium for periods longer than 7 days, the minimal time routinely used in our laboratory for scoring E^R^, resulted in the appearance of additional erythromycin-resistant colonies. In the wild-type strain, we observed after 14 days less than a 2-fold increase in the frequency of E^R^ mutations as compared to that estimated after 7 days (Table 2). In the *rad27*Δ mutant, the frequency of E^R^ mutations increased by about 12 fold after the longer incubation time, as compared to that estimated after the 7-day incubation (P < 0.0001, Mann-Whitney test). The observation that E^R^ mutants accumulate progressively on selective medium during prolonged incubation times has previously been reported for *rad27*Δ mutants [20]. We designated those E^R^ mutants which arise up to the 7th day of incubation as early mutants, and those which arise after additional incubation as late mutants.

To confirm that the E^R^ phenotype is really due to a mitochondrial mutation, we determined whether the E^R^ trait could be transferred to a *rho*^*0*^ *kar1-1* strain by cytoduction. In parallel, we also crossed each mutant with a wild-type strain to obtain a heterozygous strain for meiotic segregation analysis, so that we could corroborate the nuclear location of an E^R^ mutation if it had given a negative result of the mitochondrial location by the cytoduction test. We found that all of the tested early E^R^ mutants in the wild-type and *rad27*Δ strains (41 and 35 isolates, respectively) were able to transfer their resistance during the abortive mating with the *rho*^*0*^ recipient strain, indicating the mitochondrial location of these mutations (Table 2). In contrast, only about 50% of the tested 43 late E^R^ mutants in the *rad27*Δ strain were able to transmit their resistance phenotype by cytoduction, suggesting that a large fraction of late E^R^ mutants in *rad27*Δ was due not to mitochondrial, but to nuclear mutations (Table 2). The nuclear nature of the E^R^ mutations which had failed to be transmitted with mitochondria in the cytoduction test was confirmed by the segregation analysis of spores from dissected tetrads issued from sporulation of heterozygous strains obtained in crosses between late *rad27*Δ E^R^ mutants and a wild-type strain. For those late E^R^ mutations, heterozygous strains produced tetrads in which the resistance trait segregated 2:2 (tetrads were analyzed from 11 independent heterozygous diploid strains), indicating that non-mitochondrial E^R^ mutations were nuclear and a single mutation of this type was sufficient for a cell to acquire resistance to erythromycin. Although, contrary to long-standing views in literature [20, 52], our analysis shows that at least for *rad27*Δ strains a mutation causing resistance to erythromycin cannot be automatically equated with a mutation in mtDNA, our results confirm the earlier results by Kalifa et al. [20] that the level of mitochondrial point mutations increases due to Rad27 deficiency.

**Fig 1:**
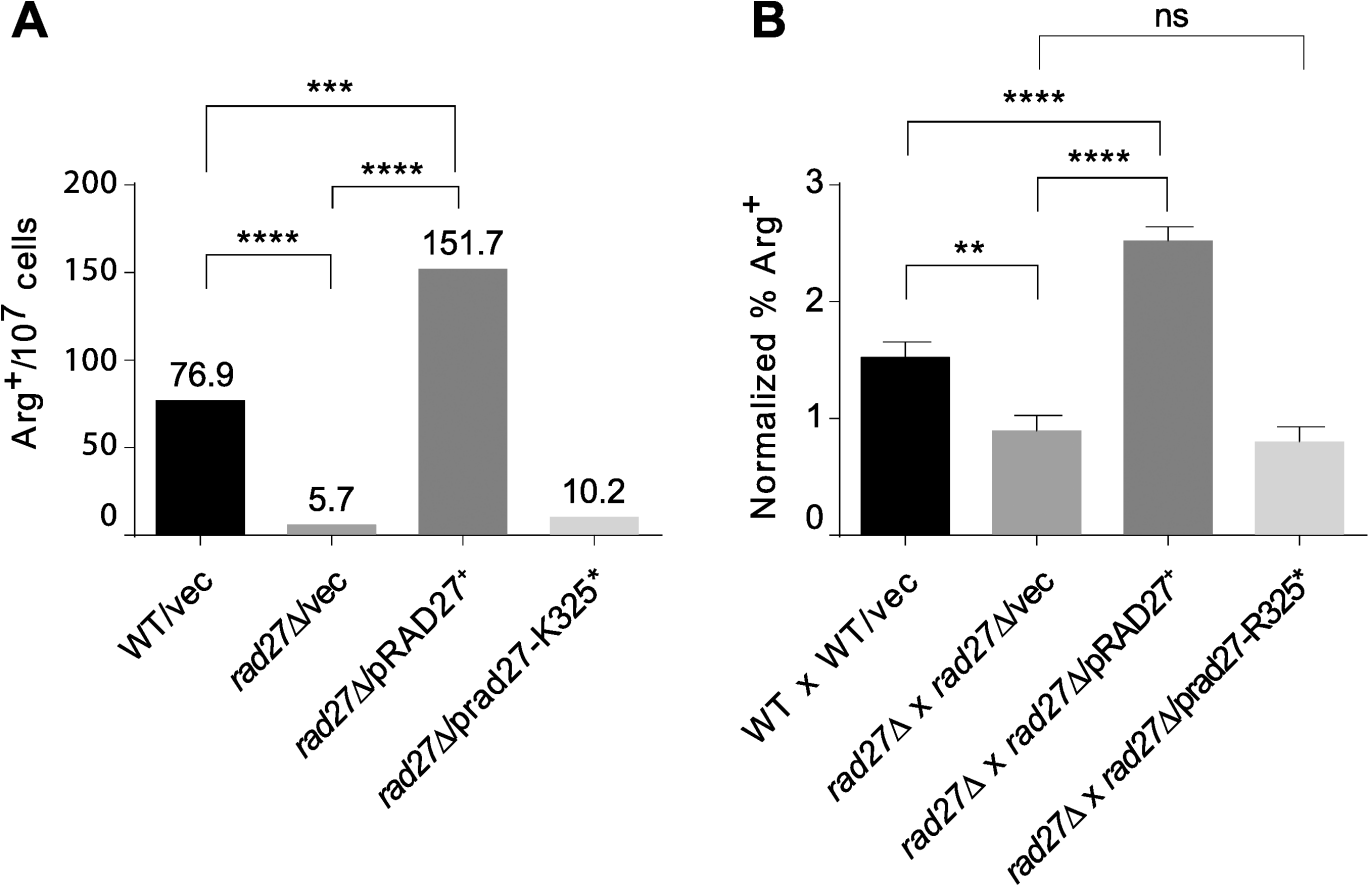
Effects of Rad27 deficiency on other mtDNA-related phenotypes. Panel **A.** Rad27 stimulates GT dinucleotide repeat instability. Frequencies of Arg^+^ prototrophs were determined in cultures of the wild-type strain (YAK136), harbouring mtDNA with the *arg8*^*m*^::(GT)_16_(+1) gene, and its *rad27*Δ derivative (YAK1629) transformed with either control vector (pRS415), p*RAD27*^+^ (pCK62) or prad27-K325* (pCK64). Values represent median frequencies in three combined independent experiments with 10–15 cultures of each transformant strain. P-values computed with the Mann-Whitney statistics–***: 0.0008; ****: < 0.0001. Panel **B**. Allelic recombination in the mitochondrial *arg8*^*m*^ reporter gene is decreased due to Rad27 deficiency. Frequencies of mitotic Arg^+^ segregants were determined in diploid cultures after crosses of wild-type strains (YAK349 xYAK243) and their *rad27*Δ derivatives (YAK1094 x YAK1097) transformed with the indicated plasmids (as in Fig 1). Values represent average frequencies in cultures obtained in 16 crosses for each pair of transformed parental strains. Error bars are SEM (standard error of the mean). P-values computed with the two-tailed unpaired t-test–**: 0.0024; ****: < 0.0001; ns: not significant.

Previous studies on the role of Rad27 in the control of stability of microsatellite sequences indicated that, in contrast to the nucleus in which Rad27 promotes microsatellite stability, inactivation of *RAD27* resulted in inhibition of rearrangements in those sequences in mitochondria [20]. However, whereas the notion that the Rad27 activity limits the stability of microsatellite sequences in the nucleus is based on several independent studies [24, 66–69], to date only one study has reported the opposite effect of Rad27 in mitochondria [20]. Therefore, we decided to verify the observation in another genetic background than the one used in the cited report. We introduced the *arg8*^*m*^::(GT)_16_(+2) reporter [26] into a *rho*^0^ derivative of our wild-type strain devoid of the nuclear *ARG8*. Subsequently, we inactivated the *RAD27* gene in the resulting strain and tested the stability of GT dinucleotide repeats in the mitochondrial *ARG8* reporter. Our results showed that deletion of the *RAD27* gene decreased over 10 fold the frequency of frameshifts within the repeated tract leading to an Arg^+^ phenotype (Fig 1A). Introduction of a low-copy-number plasmid encoding *RAD27* to *rad27*Δ cells not only compensated for the decrease, but even slightly increased the frequency of Arg^+^, confirming that Rad27 stimulates mitochondrial microsatellite instability. The plasmid containing a defective truncated allele rad27-R325*, which confers nuclear phenotypes closely resembling those exhibited by *rad27*Δ strains [13, 70], failed to suppress the increased stability of the di-nucleotide tract in the mitochondrial reporter, further corroborating the requirement of the fully functional Rad27 nuclease in regulation of mtDNA sequence stability.

Frequency of homologous recombination has also been reported to be affected by Rad27 deficiency in opposite ways in the nucleus and mitochondria. Cells lacking Rad27 exhibit a mitotic hyper-recombination phenotype [15, 71]. In contrast, the mitochondrial intra-chromosomal recombination, measured as the frequency of deletions of the intervening sequence between a 96 bp-long direct repeats, is limited by Rad27 deficiency [20]. To get broader insight into the role of Rad27 in the mitochondrial recombination, we used an *ARG8*^m^ recombination assay previously developed in our laboratory to measure the frequency of mitochondrial allelic recombination [28]. Wild-type or *rad27*Δ parental strains carrying mitochondrial heteroalleles of the *ARG8*^m^ reporter gene were crossed and frequencies of arginine prototrophs were scored in final diploid cultures. The results showed that Rad27 deficiency caused a significant decrease (p = 0.0024) in the frequency of Arg^+^ recombinants (Fig 1B), indicating that mitochondrial inter-chromosomal recombination is also dependent on the active Rad27 nuclease. The conclusion was confirmed by complementation of *rad27*Δ-dependent recombination frequency decrease within *ARG8*^m^ with the plasmid containing the wild-type *RAD27* gene and lack of complementation with the plasmid carrying the truncated rad27-R325* allele. The similar effects of *RAD27* inactivation on both intra- (between direct repeats) and inter-chromosomal recombination of mtDNA (heteroallelic) further support the general conclusion that, in contrast to its role in recombination in the nucleus, Rad27 activity stimulates mitochondrial homologous recombination.

### Inactivation of Rad27 alters the spectrum of mitochondrial mutagenesis

The mechanism underlying the enhanced mitochondrial mutagenesis conferred by Rad27 deficiency is not understood [20]. To get more insight into the mitochondrial mutator phenotype caused by the lack of the Rad27 nuclease, mutations arising in mtDNA of *rad27*Δ deletion mutants were subjected to a detailed analysis. Because individual mutagenic mechanisms often demonstrate their signatures in DNA sequence [72–74][75], the region from the position 1731 to 2293 (the coordinates of the 21S rRNA locus sequence in Saccharomyces Genome Database [55]) of the mitochondrial 21S rRNA gene was sequenced from independently isolated early and late E^R^ colonies. This region contains a stretch of three nucleotides (GAA, positions: 1957–1959) whose mutations commonly confer resistance to erythromycin [54, 62–64]. The sequence analysis of E^R^ mutations indicated that the majority of early mutations found in the wild-type strain and in the *rad27*Δ single mutant were AT → GC transitions and AT → TA transversions at position 1958 (Table 3). Those two mutations were frequent also in late mitochondrial E^R^ in the mutant strain, but, mainly, a remarkable GC → AT increase at position 1957 was found (the frequency of 19.1*/* 10^7^ *rho*^+^ in *rad27*Δ vs. 0.41*/* 10^7^ *rho*^+^ in the wild-type; Table 3).

**Table 3:** Nucleotide changes in the sequenced region of the mitochondrial 21S rRNA gene (*rib3* locus).

Position/mutation	% of mutations (number) frequency (E^R^/10^7^ *rho*^+^)			
	Early E^R^		Late E^R^	
	WT	*rad27*Δ	WT	*rad27*Δ
1955/				
G insertion	0	2.0 (1) **0.10**	4.5 (1) **0.08**	0
**1957/**				
GC→ AT	5.8 (4) **0.12**	20.4 (10) **1.03**	22.7 (5) **0.41**	73.7 (14) **19.1**
GC→ CG	0	0	22.7 (5) **0.41**	0
**1958/**				
AT→ TA	20.6 (14) **0.41**	40.8 (20) **2.07**	9.1 (2) **0.17**	15.8 (3) **4.10**
AT→ GC	67.6 (46) **1.36**	24.5 (12) **1.24**	27.2 (6) **0.49**	5.3 (1) **1.38**
AT→ CG	2.9 (2) **0.06**	8.2 (4) **0.41**	13.6 (3) **0.25**	0
**1959/**				
AT→ TA	0	0	0	5.3 (1) **1.38**
AT→ GC	2.9 (2) **0.06**	4.1 (2) **0.21**	0	0

The positions of the most common E^R^ mutations in the mitochondrial 21S rRNA gene are marked in bold. Three values are given for each mutation: the percentage of mutants with this mutation among sequenced mutants, the number of sequenced mutants with the mutation (in parentheses), the frequency of the mutation (in bold). The frequencies of mutations were calculated from the determined percentage of the mutation occurrence in the sequenced set and the frequency values in Table 2. The frequencies of mitochondrial late E^R^ (appearing between the 1^st^ score after 7 days of incubation and the 2^nd^ score a week later) were obtained by subtracting values of early mitochondrial E^R^ mutations from values of combined mitochondrial E^R^ scored after 14 days.

Since the GC → AT transition at position 1957 appeared to be a hallmark of late E^R^ mutations arising in the *rad27*Δ strain, we asked about the origin of these mutations. Theoretically, GC → AT mutations could have occurred in pre-cultures (in this case, in pre-cultured colonies) before plating cells on the selective medium and then appeared with a delay on erythromycin plates because of a slower growth rate. In point of fact, we did notice some heterogeneity of colony sizes among strains growing on the erythromycin-containing medium. Alternatively, the characteristic GC → AT mutations could arise as post-plating mutations in cells exposed to the erythromycin-mediated toxic stress. In an attempt to distinguish between these two possibilities, we measured the growth rates of a GC → AT mutant and compared them to those of AT → GC and AT → TA mutants, both in the wild-type and *rad27*Δ background. The growth test was performed by measuring average colony sizes of the mutant strains after a 4-day incubation at 28°C on the erythromycin medium. Table 4 shows that GC → AT mutants, both in the wild-type and *rad27*Δ background, exhibited a significant decrease in the average colony size as compared to those of AT → GC (P-values for comparisons of colony diameters for both the wild-type and *rad27*Δ were < 0.0001 by the two-tailed unpaired t-test) or AT → TA mutants (P-values, as above). On the other hand, a similar analysis of mutant colonies growing on YPG plates without the addition of erythromycin showed that in general there were no significant differences between colony sizes of all three mutants in both the wild-type and *rad27*Δ. Thus, the GC → AT mutation at position 1957 in the 21S rRNA gene confers a growth defect only on YPG medium in the presence of erythromycin, suggesting that the substitution at this gene position is suboptimal for the expression of the erythromycin resistance phenotype. The slower growth rate of the GC→AT mutants goes some way towards explaining the heterogeneity of observed colony sizes on erythromycin-containing media and may also be a contributing factor in delaying the growth of some pre-plating mutants.

**Table 4:** Average colony sizes of different mitochondrial E^R^ mutants.

Strain/ E^R^ mutation	AT→GC pos. 1958	AT→TA pos. 1958	GC→AT pos. 1957
**YPG + erythromycin**			
WT	229 (± 8.7)	223 (± 5.4)	87 (± 2)
*rad27*Δ	207 (± 3.7)	253 (± 7.6)	79 (± 7.6)
**YPG**			
WT	223 (± 3.1)	216 (± 3.9)	223 (± 2.2)
*rad2*7Δ	167 (± 9.3)	251 (± 4.3)	241 (± 5.6)

Measurements of colony diameters (average ± SEM) after 4 day growth of indicated strains on YPG (pH 6.2) + erythromycin or YPG (N = 25 for each data point). Strains as in Table 2. Values in arbitrary units after the Fiji software (Materials and Methods).

**Fig 2:**
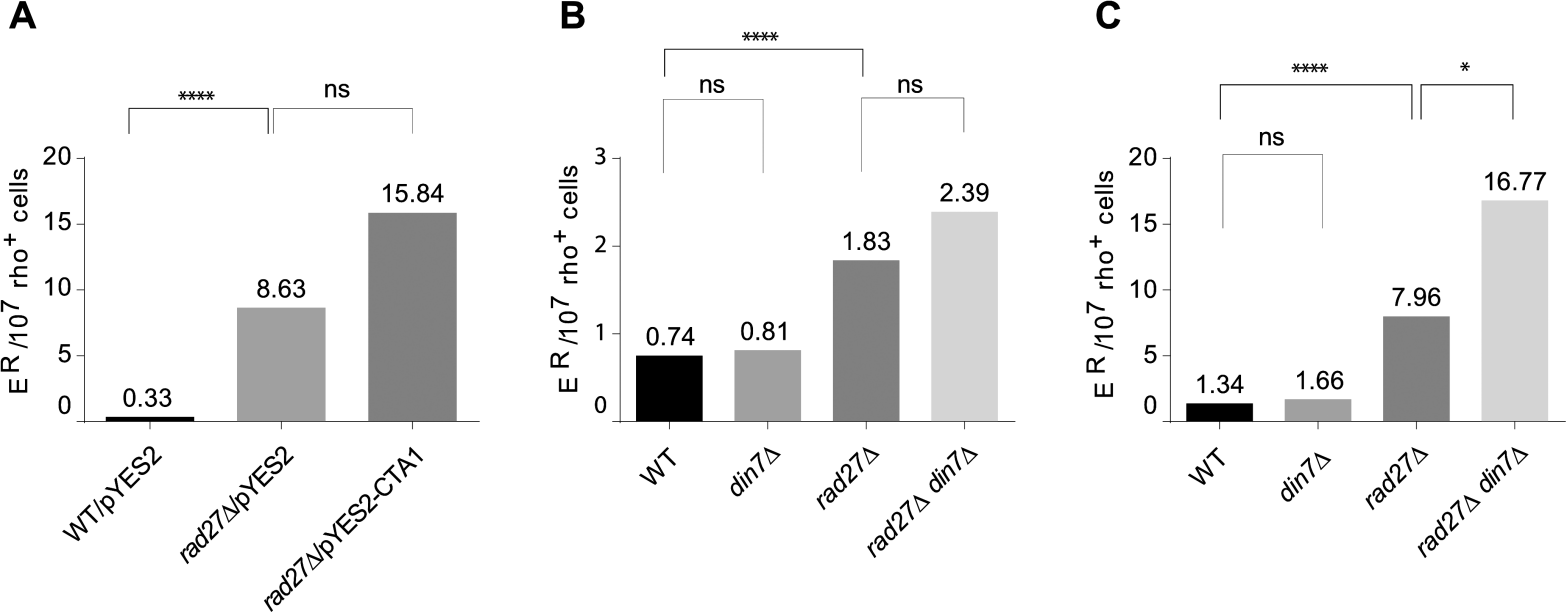
Neither oxidative stress, nor Mec1/Rad53-dependent induction of *DIN7* expression determines the increase in E^R^ mutations in Rad27 deficient yeast. Panel **A**. Overexpression of *CTA1* does not decrease the E^R^ mutation frequency in a *rad27*Δ strain. Frequency of E^R^ mutations in control strain (FF18733) and its *rad27*Δ derivative (YAK1405) transformed with indicated plasmids. The strains (10–30 independent cultures/strain) were grown overnight in a synthetic medium with casamino acids (supplemented with tryptophan as described in Materials and Methods) with 3% glycerol and 0.1% glucose (non-repressing conditions) and plated on the medium containing erythromycin. Median frequencies of E^R^ mutants scored on the 10^th^ day of incubation are shown. ns—not significant according to the Mann-Whitney statistics (P = 0.08); ****: P-value < 0.0001. Vector: pYES2; pYES2-CTA1: pCK78. Panels **B** and **C**. Enhanced mutagenesis to erythromycin resistance in a *rad27*Δ strain does not depend on the presence of Din7. The following strains: WT (FF18733), *din7*Δ (YAK1446), *rad27*Δ (YAK1441), and *rad27*Δ *din7*Δ (YAK1444) were grown overnight in YPD medium and tested on medium containing erythromycin as described in Materials and Methods. E^R^ mutants were scored on the 7^th^ (Panel **B**) and on the 12^th^ (Panel **C**) day of incubation at 28°C. Median frequencies are shown from combined three independent experiments with 10–12 cultures of each strain. ****: P-value < 0.0001; *: P-value: 0.0343; ns: not significant.

GC→AT transitions are frequently caused by either spontaneous deamination of cytosine and generation of pro-mutagenic G:U mispairs [76], or by oxidative deamination of cytosine to 5-hydroxyuracil and uracil glycol [77]. Consequently, GC→AT transitions are considered to be common mutations resulting from oxidative damage to DNA [78]. We considered the possibility that the frequent generation of GC→ AT transitions in *rad27*Δ may be related to oxidative stress-induced lesions in mtDNA. To verify this hypothesis, we examined whether the generation of E^R^ mutants in *rad27*Δ is altered in cells harbouring a multicopy plasmid bearing the *CTA1* gene. The *CTA1* gene encodes catalase A, a well-known scavenger of H_2_O_2_. Catalase A localizes both to peroxisomes and mitochondria and a significant import of catalase A into mitochondria occurs when cells are grown under respiratory conditions [79]. We have previously shown that introduction of a multicopy plasmid encoding *CTA1* suppresses antimycin A-induced rearrangements of mtDNA [35]. As shown in Fig 2A, overproduction of catalase A did not decrease the level of E^R^ mutations occurring in the *rad27*Δ strain, suggesting that the mitochondrial mutations in Rad27 deficient yeast did not originate from stress-induced oxidative lesions in mtDNA that could be counteracted by overproduction of catalase A. Consistently, we did not detect significantly increased levels of reactive oxygen species (ROS) in *rad27*Δ strains, using 2′,7′-dichlorofluorescein as a probe for ROS detection (S1 Fig).

### Mutagenesis leading to erythromycin resistance in strains lacking Rad27 does not depend on Din7

It has been shown that activation of the conserved Mec1/Rad53 cell-cycle checkpoint (called the DNA damage response; DDR in short) pathway due to instability of the nuclear DNA leads to the increased copy number of mitochondrial DNA [80]. Since Rad27 deficiency results in replication stress [15, 17, 81] and activation of checkpoint kinases [16, 17], we considered the possibility that the mitochondrial mutator phenotype of *rad27*Δ may result from activation of the cell-cycle checkpoint pathway. It is known from our previous studies that activation of the Mec1/Rad53 pathway results in increased cellular abundance of the uniquely mitochondrial nuclease Din7 [82, 83]. Furthermore, we have also shown that enhanced production of Din7 results in increased mitochondrial mutagenesis [43, 83, 84]. Thus, the mutator phenotype of *rad27*Δ strains could reflect the elevation of the Din7 nuclease level due to Mec1/Rad53 activation in Rad27 deficient yeast. To test this possibility, we compared the frequency of E^R^ mutants generated in the *rad27*Δ *din7∆* double mutant strain to that of the *rad27∆* single mutant strain. As shown in Fig 2B, early E^R^ frequencies were similar in both mutants, indicating that enhanced mitochondrial mutagenesis in cells lacking Rad27 is not dependent on the elevated level of Din7. Furthermore, after a prolonged incubation time the median frequency of E^R^ mutants in the *rad27∆ din7∆* double mutant strain increased slightly over that in the *rad27∆* single mutant strain (Fig 2C), though with a marginal statistical significance, pointing to a weak synergistic effect between deletions of *RAD27* and *DIN7*. Altogether, the results clearly show that Din7 activity is not required for the E^R^ mutagenesis in strains lacking the Rad27 nuclease.

### Dun1-dependence of mitochondrial mutagenesis in strains lacking Rad27

The major target of activated Mec1/Rad53 pathway is the pool of DNA synthesis precursors. Since yeast cells carrying the *rad27∆* mutation require both Mec1 and Rad53 to survive, we analyzed the role of Dun1, the effector kinase of the checkpoint phosphorylation cascade, in the mitochondrial mutagenesis caused by Rad27 deficiency. We constructed a *rad27∆ dun1∆* double mutant strain and established the frequency of Can^R^ and E^R^ mutations. Intriguingly, the results indicated that deletion of *DUN1* in *rad27∆* cells suppressed both nuclear and mitochondrial mutator phenotypes, resulting in an over 80% decrease of Can^R^ and an over 95% decrease of both early and late E^R^ mutations (Table 5).

**Table 5:** The effect of Dun1 and/or Sml1 deficiencies on the nuclear and mitochondrial mutagenesis in *rad27*Δ cells.

Strain/genotype	E^R^/10^7^ *rho*^+^ on 7^th^ day		E^R^/10^7^ *rho*^+^ on 14^th^ day		Can^R^/10^6^
	(fold)		(fold)		(fold)
WT	0.69		1.32		0.85
*dun1*Δ	0.70		1.38		0.74
	(1.0)		(1.0)		(0.9)
*dun1*Δ *sml1*Δ	0.3		1.69		0.74
	(0.4)		(1.3)		(0.9)
*rad27*Δ	2.33	(mt) 1.28	99.7	(mt) 27.9	179.7
	(3.4)	(1.9)	(76)	(21)	(211)
*rad27*Δ *dun1*Δ	0.72	(mt) 0.29	5.24	(mt) 1.43	23.09
	(1.0)	(0.4)	(4)	(1.1)	(27)
*rad27*Δ *dun1*Δ *sml1*Δ	2.92	(mt) 2.10	36.63	(mt) 6.87	283.2
	(4.2)	(3)	(28)	(5)	(333)

Cultures of strains with indicated genotypes: WT (FF18733), *dun1*Δ (YAK1671), *dun1*Δ *sml1*Δ (YAK1696), *rad27*Δ (YAK1441), *rad27*Δ *dun1*Δ (YAK1682) and *rad27*Δ *dun1*Δ *sml1*Δ (YAK1752), were grown overnight in YPD medium and plated on YPG (pH 6.2) medium with erythromycin or on a synthetic minimal glucose medium with canavanine. The values represent median E^R^ or Can^R^ frequencies calculated from combined results of several separate experiments with 10–15 independent cultures of each strain. To correct E^R^ frequencies in *rad27*Δ and *rad27*Δ *dun1*Δ strains for mitochondrial mutations, control cytoduction experiments were carried out to establish corresponding correction factors. Corrected values are preceded by “(mt)”. Relative changes in frequencies of E^R^ mutations in respect to those determined for the wild-type strain are shown in parentheses (fold). Since during the course of this study overall we have detected nuclear E^R^ mutations in the wild-type strain only sporadically, we assumed that all E^R^ mutations in the reference strain had been mitochondrial. P-values by the Mann-Whitney statistics of all differences between median frequencies (both E^R^ and Can^R^ mutagenesis) established for *rad27*Δ and *rad27*Δ *dun1*Δ are < 0.0001. Likewise, P-values for all the differences between *rad27*Δ *dun1*Δ *sml1*Δ and *rad27*Δ *dun1*Δ are < 0.0001. On the other hand, the difference between *rad27*Δ and *rad27*Δ *dun1*Δ *sml1*Δ in respect to early E^R^ mutagenesis is insignificant (E^R^ mutagenesis on the 7^th^ day: P-value of 0.50), whereas in respect to late E^R^ mutagenesis the difference between the same strains becomes significant: the P-value is 0.0032. The difference between the same strains in the Can^R^ mutagenesis is significant, because the computed P-value is < 0.0001.

Activation of Dun1 kinase by the Mec1/Rad53 pathway causes transcriptional induction of the ribonucleotide reductase (*RNR*) genes [85] as well as phosphorylation and concomitant degradation of Sml1, the inhibitor of the large subunit of ribonucleotide reductase, Rnr1 [86]. Consistently, in a recent report by Hendry et al. [16], the authors propose that Rnr3 abundance can be used as a specific read-out of the DDR pathway activation and genome instability (similar results were also reported earlier by Tang et al. [87]). A strain lacking Rad27 displays a highly increased abundance of Rnr3 both in spontaneous conditions (2^nd^ rank among over 5000 mutants) and when tested in the presence of a DNA-methylating agent, methylmethane sulfonate (1^st^ rank) [16]. *RNR3* transcription is repressed in unstressed cells by Crt1/Rfx1 repressor which is phosphorylated by the Dun1 kinase upon its activation by the DDR Rad53 kinase [88]. Hyperphosphorylation of Crt1/Rfx1 releases the repressor from DNA which allows the induction of target gene transcription. Thus, the increased abundance of Rnr3 in *rad27*Δ cells strongly suggests that the Mec1/Rad53 pathway is activated due to nuclear genome instability in those cells. Consequently, the activation of the signaling pathway should lead to an increase in dNTP synthesis. Elevation of dNTP pools, in turn, could result in increased mutagenesis of both nuclear and mitochondrial genomes. To check this possibility, we inactivated the *SML1* gene in the *rad27*Δ *dun1*Δ double mutant background. We expected that deletion of *SML1* should at least partially restore dNTP pools and mutator phenotypes in the *rad27*Δ *dun1*Δ strain. The prediction turned out to be correct because elimination of the ribonucleotide reductase inhibitor from *rad27*Δ *dun1*Δ cells brought back elevated levels of nuclear (Can^R^) and mitochondrial (E^R^) mutations (Table 5). This result confirms the hypothesis that both nuclear and mitochondrial mutator phenotypes in Rad27 deficient cells result from the increased dNTP pools that are a consequence of Mec1/Rad53/Dun1 pathway activation. Interestingly, however, whereas the moderate early E^R^ mutagenesis in the triple deletion *rad27*Δ *dun1*Δ *sml1*Δ mutant reached exactly the level of that measured in the *rad27*Δ mutant, the late E^R^ mutations are significantly less frequent (by 2.7 fold) in cultures of the triple deletion *rad27*Δ *dun1*Δ *sml1*Δ mutant than those detected in cultures of the *rad27*Δ single mutant. On the contrary, the level of Can^R^ mutagenesis in the *rad27*Δ *dun1*Δ *sml1*Δ strain is slightly, though significantly, higher than that in the *rad27*Δ strain. Thus, although our results point to an increase of dNTP pools as an actual source of mutagenesis in *rad27*Δ, this increase does not provide an explanation for all of the Dun1-dependent mutations in mtDNA. There is yet another Dun1-dependent mutagenic mechanism affecting mitochondrial DNA, which remains to be established.

**Fig 3:**
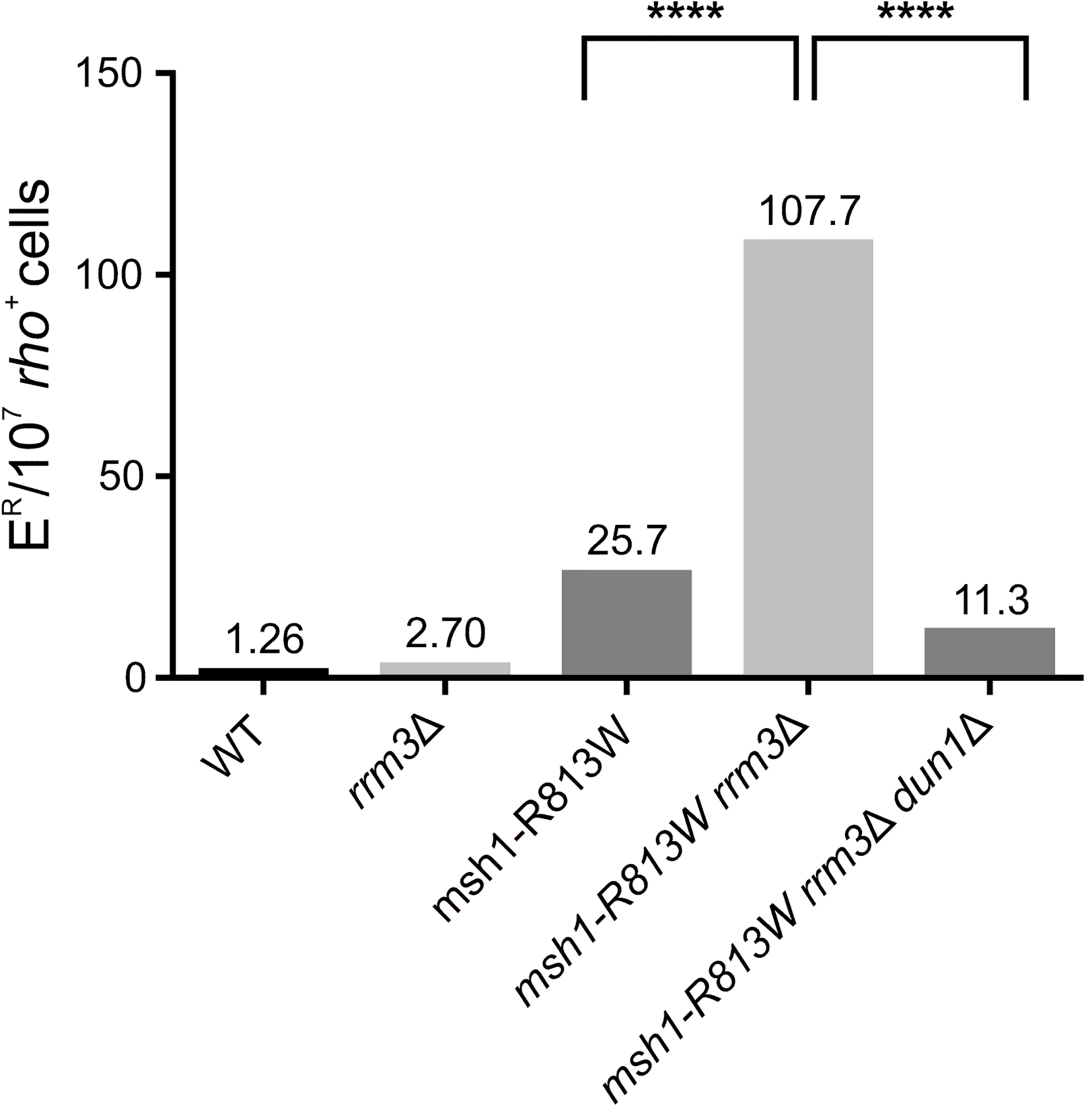
Inactivation of the *DUN1* gene suppresses the increased mitochondrial mutagenesis in an *msh1-R813W rrm3*Δ strain. The following strains: WT (FF18733), *rrm3*Δ (YAK1339), *msh1-R813W* (YAK1445), *msh1-R813W rrm3*Δ (YAK1337), and *msh1-R813W rrm3*Δ *dun1*Δ (YAK1884) were grown overnight in YPD medium and tested on the medium containing erythromycin as described in Materials and Methods. E^R^ mutants were scored on the 14^th^ day of incubation at 28°C. Median frequencies are shown from combined two to five independent experiments with 10–12 cultures of each strain. ****: P-value < 0.0001.

To find out if mutations in other repair genes have a similar to *rad27*Δ indirect effect on mitochondrial DNA, we combined a *DUN1* deletion allele with a deletion of the *RRM3* gene coding for the Rrm3 DNA helicase involved in rDNA replication [89]. Rrm3 deficiency induces *RNR3* expression, albeit at a lower level than that induced by the lack of Rad27 activity [16]. On the other hand, it has previously been reported that inactivation of *RRM3* results in increased levels of mitochondrial point mutations [90]. Surprisingly, in our strains lack of Rrm3 resulted in only a slight (2-fold), though statistically significant, increase of E^R^ mutation levels (Fig 3), suggesting that the previously described effect of *rrm3*Δ on mitochondrial DNA could be dependent on the specific background of the strain under study and/or on the capacity of mitochondrial DNA repair machinery. Therefore, we analyzed the *rrm3*Δ-induced mitochondrial mutagenesis in yeast strains harbouring a deficient allele of the *MSH1* gene encoding the mitochondrial DNA repair protein [91][28]. The R813W mutation in the ATPase domain of the Msh1 protein increased the frequency of E^R^ mutations by 20 fold in the *RRM3*^+^ context, but in the *rrm3* null background the *msh1* allele caused a synergistic effect increasing the frequency of E^R^ by 85 fold (Fig 3). This result confirms that the detection of *rrm3*Δ effect on mtDNA stability depends on the mtDNA repair potential and suggests that Msh1 is involved in the repair of *rrm3*Δ-provoked DNA lesions in this compartment. Dun1 deficiency entirely abolished the synergistic effect of *rrm3*Δ *msh1-R813W* double mutation on E^R^ mutation frequency (Fig 3). The results indicate that *rrm3*Δ-induced mitochondrial point mutations are Dun1-dependent, which suggests in turn that the indirect effect of Dun1 activation on mtDNA stability concerns not only cells lacking Rad27, but generally also other cells in which the DNA damage checkpoint pathway is activated. This suggestion, however, requires further investigations.

### The effect of Exo1 overproduction on mitochondrial phenotypes resulting from Rad27 deficiency

The results showing that Dun1 activation-dependent activities are responsible for mitochondrial mutagenesis in *rad27*Δ mutant points to the perturbation of nuclear DNA metabolism in this mutant as the main cause of the mitochondrial mutator effect. To verify this idea, we suppressed the nuclear effects of *rad27*Δ by overproducing Exo1. Exo1 is a 5’-3’exonuclease and a flap endonuclease engaged in DNA recombination and several other DNA repair pathways [92–94]. It was previously reported that overproduction of Exo1 leads to suppression of nuclear phenotypes connected with Rad27 deficiency [13, 17, 49, 95–97]. We analyzed the effect of *EXO1* overexpression under control of the *GAL1* promoter on the frequency of E^R^ in the *rad27*Δ background. As a control, we used an isogenic construct overexpressing *RAD27* and, in parallel, we monitored the effect of overproduction of both nucleases on the level of nuclear mutagenesis (Can^R^). As shown in Table 6, the nuclear and mitochondrial mutator phenotypes are suppressed to the same extent due to Exo1 overproduction.

**Table 6:** Overexpression of *EXO1* suppresses both the mitochondrial and nuclear mutator phenotypes in *rad27*Δ cells.

Strain/plasmid	E^R^/10^7^ *rho*^+^ on 7^th^ day		E^R^/10^7^ *rho*^+^ on 14^th^ day			Can^R^/10^6^
	(fold)		(fold)			(fold)
WT/ vec	0.22	(mt) 0.22	0.45		(mt) 0.45	0.32
*rad27*Δ/ vec	2.53	(mt) 1.18	36.0		(mt) 10.4	46.60
	(11.5)	(5.4)	(80)		(23)	(146)
*rad27*Δ/ pGAL1-RAD27	0.40		1.15			1.55
	(1.8)		(2.6)			(4.7)
*rad27*Δ/ pGAL1-EXO1	0.54	(mt) 0.32	3.43	(mt) 0.69		7.66
	(2.4)	(1.4)	(7.6)	(1.5)		(24)

The strains were cultured in a medium selective for the presence of plasmids with galactose as the sole carbon source and tested as described in Materials and Methods. The values represent median E^R^ or Can^R^ frequencies calculated from combined results of 3 separate experiments with 10–15 independent cultures of each strain. E^R^ isolates (2 per plate) of both WT (FF18733) and *rad27*Δ (YAK1441) with the control plasmid and *rad27*Δ with pGAL1-EXO1 were analyzed by a cytoduction test for the localization of E^R^ mutations in mtDNA. The results of the test were used to compute a correction factor for mitochondrial mutations, as in Table 5. Corrected values are preceded by “(mt)”. The construct pGAL1-EXO1 is pYES-DEST52-EXO1-V5-6xHis and pGAL1-RAD27 is pYES-DEST52-RAD27-V5-6xHis [48]. P-values, calculated using the Mann-Whitney statistics, for differences between levels of E^R^ mutations (at both time points) and Can^R^ in *rad27*Δ with the control plasmid vs. *rad27*Δ with pGAL1-EXO1 were < 0.0001.

**Fig 4:**
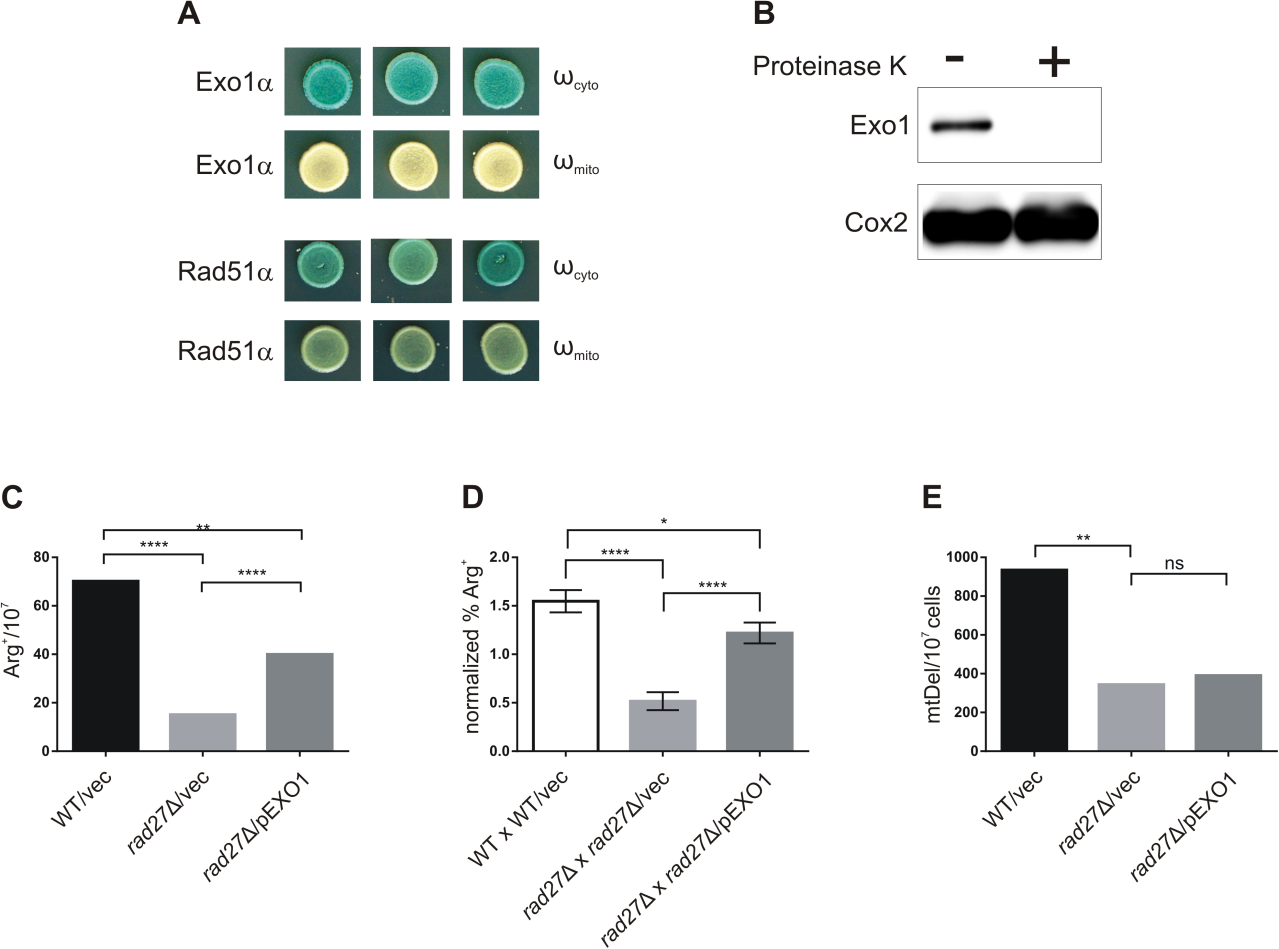
Overproduced Exo1 indirectly suppresses most of the mitochondrial phenotypes of Rad27 deficient strains. Panels A and B. Exo1 is not localized to mitochondria in yeast cells. Panel **A**. Cultures of the wild-type haploid strain (FF18733) co-expressing indicated α fusions with a cytosolic ω fragment (ω_cyto_) or ω attached to a mitochondrial targeting sequence (ω_mito_) were spotted on galactose medium plates containing X-gal and incubated for 5 days at 28°C. Panel **B**. Mitochondrial localization of Exo1-6xHis was verified by fractionation of cells from a galactose grown culture of a *rad27*Δ strain harbouring the pGAL1-EXO1 plasmid (the same strain was tested in experiments presented in Table 6). Equal portions of the resulting mitochondrial fraction were or were not treated with proteinase K and analyzed by Western blotting using an anti-His antibody for the detection of the Exo1 protein and an anti-Cox2 antibody for the detection of the control Cox2 protein localized in the inner mitochondrial membrane. Panels **C-E** The effects of *EXO1* overexpression on mitochondrial phenotypes caused by Rad27 deficiency. Panel **C**. The frequency of arginine prototrophs arising by rearrangements of mitochondrial microsatellite sequences in strains transformed with control vector (YEp13) or pRDK480 overexpressing *EXO1* (pEXO1) and bearing in their mtDNA the *ARG8*^m^ gene interrupted out-of-frame by a tract of GT dinucleotide repeats. The values represent medians of Arg^+^ frequencies in cultures grown in a glucose selective medium from three combined separate experiments with 10–15 independent cultures/transformant strain. P-values were computed using the Mann-Whitney statistics. **: 0.003; ****: < 0.0001. WT: YAK136; *rad27*Δ: YAK1629. Panel **D**. Mitochondrial allelic recombination within the *ARG8*^m^ reporter gene in crosses of wild-type strains (YAK349 xYAK243) and their *rad27*Δ derivatives (YAK1094 x YAK1097) transformed with indicated plasmids (the same as in panel A). Frequencies of Arg^+^ prototrophs were determined in diploid cultures grown under conditions selective for the presence of the plasmids. The columns correspond to average values of Arg^+^ frequencies in cultures obtained in 16 crosses for each pair of parental strains. Error bars are SEM. P-values were computed using the two-tailed unpaired t-test. *: 0,0462; ****: < 0.0001. Panel **E**. Levels of direct-repeat mediated deletions in mtDNA of a reference strain (EAS748) and its *rad27*Δ derivative (YAK1659) transformed with a vector plasmid (as above) and pRDK480. The columns represent median frequencies, from two combined separate experiments with 10–20 colonies/transformant strain, of respiring cells arising after the *ARG8*^*m*^ deletion in mtDNA in 4 day-old colonies of indicated transformant strains, grown on plates with a synthetic complete medium selective for the presence of the plasmid (without leucine), but supplemented with arginine. P-values by Mann-Whitney statistics: **: 0.003; ns: not significant.

Exo1 is considered a cytoplasmic/nuclear protein and as far as we know no report has indicated its entrance into mitochondria. However, since in our experiments Exo1 was overproduced, we wanted to make sure that the Exo1-mediated suppression was not due to the possible moonlighting activity of this nuclease in mitochondria. We employed a previously described *lacZ* α-complementation assay for dual protein localization in yeast cells [47, 59] and constructed a plasmid encoding a fusion protein in which the α fragment of β-galactosidase was attached to the C-terminus of Exo1. The Exo1-α fusion protein was produced under the control of the *GAL1* promoter. This plasmid was co-expressed in yeast cells with an ω fragment of β-galactosidase localized either to the cytoplasm (ω_cyto_/ ω_c_) or mitochondria (ω_mito_/ ω_m_). As shown in Fig 4A, the β-galactosidase activity was detected in a plate assay in yeast strains which beside the Exo1-α protein fusion produced ω_c_, but not ω_m_. In contrast, we obtained positive results of the α-complementation assay for both ω_c_ and ω_m_ when we tested the reference Rad51-α fusion protein, which could be expected to reside both in the cytoplasm and mitochondria according to a recent report by Stein et al. [98]. The absence of Exo1 from mitochondria was additionally confirmed by fractionation of *rad27*Δ cells harbouring pGAL1-EXO1 and analysis of resulting mitochondrial fractions with proteinase K followed by Western blotting (Fig 4B). Although we detected Exo1 in the crude fraction containing mitochondria and other cellular membranes, the Exo1 band disappeared after treatment of the fraction with proteinase K. Altogether, the results confirm that the full length Exo1 does not enter mitochondria and that the overproduction of Exo1 in the nucleus is sufficient to suppress mitochondrial E^R^ mutagenesis resulting from Rad27 deficiency.

This result raised the question of how the Exo1 overproduction affects other mitochondrial phenotypes caused by Rad27 deficiency. We introduced a multicopy plasmid pRDK480 [49] containing an insert with the *EXO1* gene into cells carrying the mitochondrial *ARG8*^m^ gene interrupted by the GT_16_ (+2) tract. As a control, to verify the ability of Exo1 overproduction to suppress the nuclear mutagenesis in Rad27 deficient cells in the background of the Arg^¯^ non-respiring strain, instead of using the standard canavanine-resistance assays, we tested the cycloheximide-resistance mutagenesis as an indicator of accumulating point mutations in the nuclear genome (as used previously in e.g. [99]). Introduction of this plasmid into *rad27*Δ cells caused an over 90% decrease in the level of nuclear mutations conferring resistance to cycloheximide (S2 Table), confirming the expected suppression effect. The overexpression of *EXO1* also significantly increased the level of mitochondrial microsatellite instability (Fig 4C), indicating that suppression of DNA instability in the nucleus affects microsatellite stability in mitochondria of *rad27*Δ cells. However, although we noticed a significant increase in Arg^+^ frequency due to the presence of pRDK480, this increase did not reach the Arg^+^ level measured in cells producing the wild-type Rad27 protein. This partial effect suggests that the fraction of Rad27 residing in mitochondria can contribute to the stimulation of mitochondrial microsatellite instability as well. In the allelic recombination assay with Exo1-overproducing *rad2*7Δ cells, over 70% of this recombination was restored by introduction of pRDK480 into *rad27*Δ cells (Fig 4D). On the basis of the results, we cannot exclude that the presence of Rad27 in mitochondria also directly influences allelic recombination of mtDNA. However, this effect seems to be marginal, since the full suppression effect could be expected at the level of 90%. Altogether, the result indicates that nuclear processes activated in *rad2*7Δ cells are responsible for limiting a great part of mitochondrial allelic recombination. Curiously, the overproduction of Exo1 in yeast cells, carrying a previously described system of direct repeats in mtDNA [20, 34], did not affect the level of direct repeat-mediated deletions (DRMD) in the *rad27*Δ mutant (Fig 4E). This result suggests that, in contrast to the above-mentioned allelic recombination, mitochondrial Rad27 functions directly in the homologous recombination pathway leading to DRMD in mtDNA.

## Discussion

Our analysis of mitochondrial phenotypes resulting from Rad27 deficiency revealed a complexity of mechanisms affecting the stability of mitochondrial DNA in the mutant cells. First, we established that three aspects of modified mitochondrial genome stability in *rad27*Δ cells: the level of point mutations conferring resistance to erythromycin, allelic recombination and the stability of mitochondrial microsatellite sequences, are either entirely (mitochondrial E^R^ mutations), predominantly (allelic recombination) or partially (microsatellite stability), indirect consequences of the nuclear DNA replication defect in those cells. On the other hand, we point to homologous recombination between direct repeats as the target of mitochondrial activity of Rad27. Interestingly, whereas the absence of Rad27 in mitochondria causes the decrease in the frequency of DRMD in mtDNA, the absence of Rad27 in the nucleus leads to a hyper-recombination phenotype in nuclear DNA [23]. While the nuclear phenotype reflects an enhanced formation of recombinogenic DNA intermediates due to DNA replication problems in cells lacking Rad27, the decrease in recombination frequency in mitochondria indicates that formation of recombination intermediates is not stimulated by the absence of Rad27 in this compartment and that Rad27 activities are not important for mitochondrial replication. Instead, Rad27 stimulates a sub-pathway of mitochondrial homologous recombination. Since recombination processes in mtDNA are far less understood than those operating during recombination in the nucleus, it remains to be established which recombination mechanism engages the Rad27 activity in mitochondria. Based on previous evidence on the nuclear DNA recombination, the single-strand annealing (SSA) recombination requiring Rad52, but not Rad51, is the mechanism responsible for the majority of nuclear direct-repeat mediated deletions in the nuclear genome [100, 101]. However, the recent report by Stein et al. [98] indicated that a significant part of DRMD in mitochondria required the Rad51 activity, suggesting that gene conversion mechanisms can contribute to deletions of intervening sequences between direct repeats in mtDNA. On the other hand, nuclear DRMD events are believed to represent predominantly an intra-chromosomal recombination [102, 103]. For mitochondria, the situation is less obvious, since the mitochondrial genome exists in multiple copies per cell and the contribution of inter-chromosomal recombination into mitochondrial DRMD cannot be excluded *a priori*. However, our analysis indicating a stark difference in their requirements for mitochondrial Rad27 activity between allelic recombination, which proceeds inter-chromosomally, and DRMD supports the notion that the two mitochondrial homologous recombination pathways are mechanistically different and that mitochondrial DRMD may proceed predominantly intra-chromosomally.

In contrast to the stimulating effect on homologous recombination in mitochondria, Rad27 protects against point mutations in both mitochondrial and nuclear DNA, as the frequency of both nuclear and mitochondrial mutagenesis increases in *rad27*Δ mutants. It is worth noting that genetic analysis, using cytoduction and meiotic segregation, of frequent E^R^ mutations isolated in *rad27*Δ strains showed that these mutations originated either in the mitochondrial genome or, unexpectedly, in the nucleus. We did not attempt to identify or even characterize more closely those nuclear E^R^ mutations, but in general those mutations were becoming prevalent among E^R^ mutations only after an incubation time longer than 1 week. The timing suggests that those mutations may appear post-plating under selection conditions in the presence of erythromycin.

The previous study, based on the recognition of the Rad27/FEN1 role in the nuclear long-patch base excision repair (L-P BER) [104] and the subsequent finding that L-P BER functions in mammalian mitochondria [18, 21], strongly suggested that the mutator phenotype caused by *RAD27* deletion in yeast reflected a defect of the mtDNA repair process directly engaging the Rad27 nuclease in mitochondria [20]. Contradictory to this suggestion, our results show that the mutator effects of the *rad27*Δ mutation, both nuclear and mitochondrial, can be suppressed by overproduction of Exo1. Whereas the suppression of the nuclear mutagenesis, as well as other nuclear phenotypes, caused by Rad27 deficiency, is well documented and has been reported on many occasions [13, 17, 49, 95–97], the mitochondrial effect of Exo1 is novel and unexpected. The activity of this nuclease has not been connected with mitochondria so far and the present study has confirmed that Exo1 does not enter the mitochondrial matrix, even when overproduced. Consequently, the Exo1-mediated suppression of replication defects in nuclei of cells lacking Rad27 is sufficient to protect mitochondrial DNA against accumulating point mutations. Thus, it is not the absence of the mitochondrial Rad27 nuclease *per se* that affects the level of point mutations in mtDNA. Instead, the absence of Rad27 in the nucleus, leading to a defect in the maintenance of nuclear DNA integrity, induces the mitochondrial mutagenesis.

The analysis of the specificity of mutagenesis in the mitochondrial 21S rRNA gene (*rib3* locus) revealed that a GC → AT transition is the signature for mutagenesis leading to resistance to erythromycin induced by Rad27 deficiency. Coincidentally, the mutation at the site, adjacent to the most common site of mitochondrial E^R^ mutations, confers a slower-growing resistance phenotype. GC → AT transitions are considered to be frequent mutations resulting from the oxidative damage to DNA [78]. Additionally, earlier reports have suggested that high nuclear levels of gross chromosomal rearrangements in *rad27* or *rad52* mutants are due to oxygen metabolism [105] and, on the other hand, that deletion of *RAD52* causes an increase in the level of reactive oxygen species [39]. However, our investigations did not confirm the role of oxidative damage in mitochondrial mutagenesis resulting from Rad27 deficiency. Instead, we show the dependence of this mutagenesis on the activation of Dun1 kinase, an effector of the conserved checkpoint pathway activated in response to replication stress and DNA damage, controlled by Mec1 and Rad53. A primary endpoint of Mec1/Rad53 pathway activation is an increase in dNTP synthesis, due to the induction of the expression of *RNR* genes encoding subunits of ribonucleotide reductase [85], and degradation of an inhibitor of this enzyme, the Sml1 protein [86, 106, 107]. In a genome-wide study of Rnr3 abundance across 5200 yeast mutants, a strain lacking Rad27 has exhibited in normal growth conditions, without any exogenous challenge, a very high level (2^nd^ rank among the whole set of tested mutants) of the Rnr3 subunit [16]. The high induction of RNR subunits leads to a strong increase of dNTP pools, which is eliminated by inactivation of *DUN1* and partially restored by subsequent deletion of *SML1*. According to our results, mitochondrial mutagenesis is regulated in a manner analogous to that of Mec1/Rad53-induced dNTP levels: it is increased due to Rad27 deficiency, then it is decreased in *rad27*Δ strains upon deletion of *DUN1* and partially restored in *rad27*Δ *dun1*Δ cells upon subsequent *SML1* inactivation. This partial correlation points to the Mec1/Rad53-mediated elevation of dNTP pools as the actual source of increased mutagenesis in mitochondria of Rad27 deficient cells. On the other hand, it also suggests the existence of an additional mechanism affecting mitochondrial mutagenesis in a manner depending on Dun1 activation that is not restored in the triple deletion mutant *rad27*Δ *dun1*Δ *sml1*Δ.

The important question raised by our results is whether the induction of mitochondrial mutagenesis by Dun1 activation is a specific response to Rad27 deficiency or whether it is a manifestation of some general response to DNA replication stress. There are several lines of evidence suggesting that activation of the Mec1/Rad53 pathway modulates the stability of mitochondrial DNA. On the one hand, it has been reported that deletion of the *RRM3* gene, encoding a nuclear DNA helicase, causes both transcriptional induction of *RNR3* [16] and a mitochondrial mutator phenotype [90]. Our current results indicate that this mutagenesis is dependent on Dun1 activity. On the other hand, there is convincing evidence that activation of the Mec1/Rad53 pathway increases mtDNA copy number and, similar to the mitochondrial mutator effect in *rad27*Δ cells, this increase is dependent on increased dNTP pools [80]. Those findings together with our results support the proposal that modulation of the stability of mtDNA is one of the endpoints of the general cellular response to replication stress and activation of the Mec1/Rad53/Dun1 signaling pathway.

## Conclusions

Presented results reveal that abrogation of replication stress in the nucleus entirely or partially suppresses several mitochondrial phenotypes caused by Rad27 deficiency, namely: increased point mutations, decreased microsatellite instability and allelic recombination. These results let us conclude that nuclear DNA instability affects mitochondrial DNA stability. Consistently, we show that the mitochondrial mutator effect that increases levels of mitochondrial point mutations is initiated by nuclear DNA instability and is entirely dependent on the checkpoint kinase Dun1. We have also identified increased deoxyribonucleotide pools as one of Dun1-activated mechanisms resulting in the mitochondrial mutator phenotype. On the other hand, we also show that homologous recombination leading to deletions between direct repeats is not changed by the nuclear response and that this recombination pathway is a direct target of the mitochondrial Rad27 activity.

## Supporting information

S1 Fig ROS levels measured fluorometrically with dichlorofluoresceine diacetate in the wild-type and *rad27*Δ strain. The measurement was performed according to the procedure described previously [35]. Cells (WT: FF18733 and *rad27*Δ: YAK1405) were grown in a rich medium with glycerol as the sole carbon source. The RFU values represent relative fluorescence values measured with a Cary Eclipse fluorescence spectrophotometer (fluorescence excitation of 485 nm and emission at 520 nm) normalized for 10^6^ cells assayed. Columns represent mean values obtained from five measurements and error bars depict standard deviations.
(TIF)

S1 Table Primers used in this study. (DOCX)

S2 Table Mutations leading to cycloheximide resistance in *rad27*Δ Arg¯ strains are suppressed by overexpression of the *EXO1* gene. (DOCX)

S1 Text Additional methods. (DOCX)
