# Supplementary figures and images for: Activation of Dun1 in response to nuclear DNA instability accounts for the increase in mitochondrial point mutations in Rad27/FEN1 deficient S. cerevisiae
Source: PLoS One. 2017 Jul 5;12(7):e0180153. doi: 10.1371/journal.pone.0180153 (PMC5497989; doi:10.1371/journal.pone.0180153)

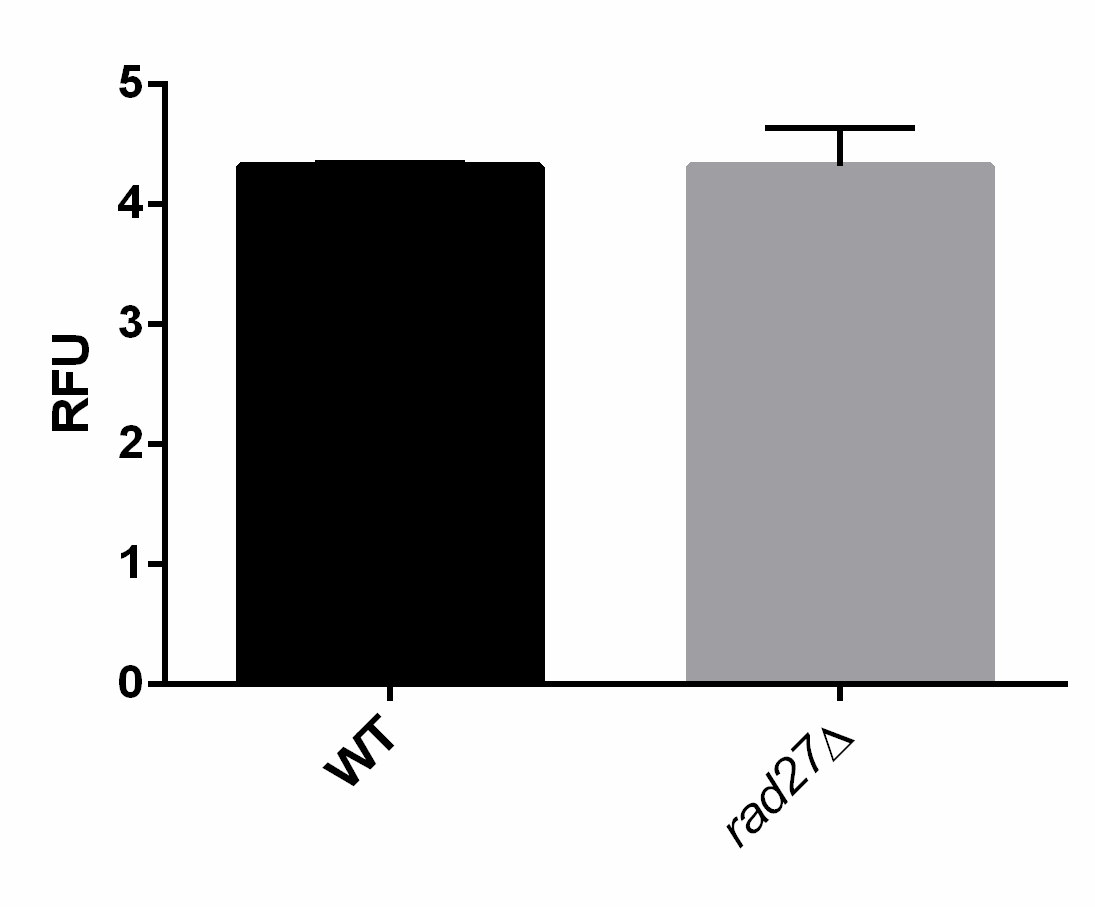

Supplement: S1 Fig — The measurement was performed according to the procedure described previously [35]. Cells (WT: FF18733 and rad27Δ: YAK1405) were grown in a rich medium with glycerol as the sole carbon source. The RFU values represent relative fluorescence values measured with a Cary Eclipse fluorescence spectrophotometer (fluorescence excitation of 485 nm and emission at 520 nm) normalized for 106 cells assayed. Columns represent mean values obtained from five measurements and error bars depict standard deviations. (TIF) [file pone.0180153.s001.tif]
