# Supplementary material for: Activation of Dun1 in response to nuclear DNA instability accounts for the increase in mitochondrial point mutations in Rad27/FEN1 deficient S. cerevisiae
Source: PLoS One. 2017 Jul 5;12(7):e0180153. doi: 10.1371/journal.pone.0180153 (PMC5497989; doi:10.1371/journal.pone.0180153)
Supplement: S1 Table — (DOCX) [file pone.0180153.s002.docx]

**S1 Table. Primers used in this study.**

| *Name* | *Sequence* | *Reference* |
| --- | --- | --- |
| OA54 | ACGCTAACATCAATTCCCATATCAA | This study |
| ARG8_D | GCACCACATGTACATATCACGTAAG | a |
| kanB | CTGCAGCGAGGAGCCGTAAT | a |
| kanC | TGATTTTGATGACGAGCGTAAT | a |
| hphC | AGCAGACGCGCTACTTCGAG | This study^b^ |
| hphB | ACTTCCGGAATCGGGAGCGC | This study^b^ |
| natC | TCGTGGTCGTCTCGTACTCC | This study^c^ |
| RAD27_A | GCTGGTAAGTTATGATAGAAAGCCA | a |
| RAD27_B | CTTCATCTTTTCCAATTCTGTTGTT | a |
| RAD27_C | CCTTCTTGTTGAGACATTTGACTTT | a |
| RAD27_D | TTAATGTTGCGTTTTGTGTTTCTTA | a |
| DIN7_A | TTGTAACATTCATTTCAAAGCACAT | a |
| DIN7_B | GCAATAATCAATAATACATTTCGCC | a |
| DIN7_C | GGGTGTAGTCACTGCTATGAAGATT | a |
| DIN7_D | ATGGTGTTATTTTGATGGTTGATTT | a |
| DUN1_A | AGAAGCCCCTGAATACCATAAATAG | a |
| DUN1_B | ATATTTTCACAGCTACTTGCTGACC | a |
| DUN1_C | GAAGTCCTCACAAAGAAAGGATACA | a |
| DUN1_D | CGATGTCAGAGATTTAGAGGAAAAA | a |
| RRM3_A | TATCTTCCCTTACCGGATTTATTTC | a |
| RRM3_B | TTAGCAGTGGTTTTATTTTCTTTGG | a |
| RRM3_C | ATAAGGAAAAATTCCCCTATGTGAG | a |
| RRM3_D | GGCTAGATCTCCTTTTTCAGTTTCT | a |
| OA152 | TAGTCGAGAGTAACAAGTAAAGG | This study |
| OA153 | TTTGAAATAATGCTTCTCATG | This study |
| 5-sml1 | CCAAACGGGCTCCACTACC | d |
| 3-sml1 | TGGCGCTAGCGATATCTAGC | d |
| SML1_A | CATATCGTTACTGTTTTGGAACATCGC | a |
| SML1_B | AGACAACATAGGAACCTCAGCCATAGGT | a |
| SML1_C | TGGAGGAGAGACTCAACTCTATCGATCA | a |
| SML1_D | TAAAGGGAAAGGAAAATGCACG | a |
| MSH1_D | TCCTTACCGTAATAAATATCCACCA | a |
| MSH1_C1 | ACTGGGCTATCTTGATACCTTATC | This study |
| OA112 | CAATCTTGATATATACCAGATT**A**AACTCTTTCTTCACTGAATTTCTTATC | This study^e^ |
| OA111 | GATAAGAAATTCAGTGAAGAAAGAGTT**T**AATCTGGTATATCAAGATTG | This study^e^ |
| OA85 | ATATACCTCTATACTTTAACGTCAAGGAGAAAAAACTATA*ATGGGTATCCAAGGTCTTCTTC* | This study^f^ |
| OA86 | ACGTTGTAAAACGACGGCCAGTGAATCCGTTTGACCCGG*TTTACCTTTATAAACAAATTGGG* | This study^g^ |
| OA68 | ATATACCTCTATACTTTAACGTCAAGGAGAAAAAACTATA*ATGTCTCAAGTTCAAGAACAAC* | This study^f^ |
| OA69 | ACGTTGTAAAACGACGGCCAGTGAATCCGTTTGACCCGG*CTCGTCTTCTTCTCTGGGGTCAC* | This study^g^ |

^a^ Saccharomyces Genome Deletion Project. RRID:SCR_014961. Available: http://www-sequence.stanford.edu/group/yeast_deletion_project/

^b^ Primer specific for the *hphMX4* deletion cassette for verification of correct integrations at deleted loci.

^c^ Primer specific for the *natMX4* deletion cassette for verification of correct integrations at deleted loci.

^d^ Dmowski M, Rudzka J, Campbell JL, Jonczyk P, Fijalkowska IJ (2017) Mutations in the Non-Catalytic Subunit Dpb2 of DNA Polymerase Epsilon Affect the Nrm1 Branch of the DNA Replication Checkpoint. PLoS Genet 13: e1006572.

^e^ The change introduced in the *RAD27* sequence, corresponding to the sequence of the *rad27-R325** allele, is marked in bold.

^f^ In italics, the sequence corresponding to the N-terminal part of *EXO1*, the rest of the sequence is homologous to the proximal to START codon part of the *GAL1* promoter. Analogous for the primer OA68 that was used for *GAL1*-*RAD51*-α construction.

^g^ In italics, the sequence corresponding to the C-terminal part of *EXO1*, the rest of the sequence is homologous to the sequence encoding a linker peptide followed by a fragment of the lacZ protein (after Karniely et al., 2006). Analogous for the primer OA69 that was used for *GAL1*-*RAD51*-α construction.
