# Supplementary material for: Activation of Dun1 in response to nuclear DNA instability accounts for the increase in mitochondrial point mutations in Rad27/FEN1 deficient S. cerevisiae
Source: PLoS One. 2017 Jul 5;12(7):e0180153. doi: 10.1371/journal.pone.0180153 (PMC5497989; doi:10.1371/journal.pone.0180153)
Supplement: S2 Table — (DOCX) [file pone.0180153.s003.docx]

**S2 Table. Mutations leading to cycloheximide resistance in *rad27*Δ Arg¯ strains are suppressed by *EXO1* overexpression.**

| Strain background | WT/ vec | *rad27*Δ/ vec | *rad27*Δ/pRDK480 | % suppression |
| --- | --- | --- | --- | --- |
| YAK136^a^ | 0.12 | 75.9 | 7.44 | 90.2 |
| EAS748^b^ | <0.3 | 33.2 | 3.2 | 90.4 |

Tests were performed as described in **Materials and Methods**. Numbers represent median frequencies of cycloheximide resistant mutants in cultures of indicated transformant strains. “vec”: strains transformed with the reference plasmid Yep13. The pRDK480 plasmid is a 2μ-*LEU2* vector with an *EXO1*-containing insert (Tishkoff, et al. [1997] Proc Natl Acad Sci U S A 94: 7487-7492).

^a^ YAK136 is a non-respiring wild-type strain, harbouring mtDNA with the *arg8^m^*::(GT)_16_(+2) reporter, and its *rad27*Δ derivative is YAK1629 (Table 6).

^b^ EAS748 (Phadnis, et al. [2005] Genetics 171: 1549-1559) is a non-respiring wild-type strain, carrying mtDNA with the Rep96::*ARG8^m^::cox2* reporter, and its *rad27*Δ derivative is YAK1659 (Table 6). For these strains, a modified procedure for estimation of cycloheximide-resistant mutant frequencies was used. Namely, 10 transformant colonies for each tested strain, growing on a selective medium, were pooled in 100 μl of sterile water. A 5-μl aliquot of the suspension was diluted appropriately in water to establish the density of transformant cells by plating on a selective medium and the rest of the suspension was plated on a medium with cycloheximide.
