# Supplementary material for: Activation of Dun1 in response to nuclear DNA instability accounts for the increase in mitochondrial point mutations in Rad27/FEN1 deficient S. cerevisiae
Source: PLoS One. 2017 Jul 5;12(7):e0180153. doi: 10.1371/journal.pone.0180153 (PMC5497989; doi:10.1371/journal.pone.0180153)
Supplement: S1 Text — (DOCX) [file pone.0180153.s004.docx]

**Additional methods**

**Cytoduction experiments**

Cytoduction experiments to establish E^R^ mutation localization were performed by crossing donor E^R^ *MAT***a** (*rho*^+^) strains, derived from FF18733 (Table 6), with the recipient *MAT***α** *kar1-1 rho*^0^ strain, DFS160 (Table 6) in YPD medium, essentially as described in [[1](#_ENREF_1)]. Cells after the crosses were diluted and plated for single colonies on a synthetic minimal glucose medium supplemented uniquely with the three requirements needed for the DFS160 strain that are not sufficient for the growth of the *MAT*a strains: leucine and arginine at replete concentrations of 217 mg/l (after [[2](#_ENREF_2)]) and 40 mg/l, respectively, and adenine at the concentration of 10 mg/l. The limited concentration of adenine allowed for the expression of the red growth phenotype of the *ade2* recipient strain and for the clear-cut discrimination between bigger and more intensively coloured colonies of rare respiring DFS160, resulting from the cytoduction, and the majority of smaller and paler non-respiring DFS160 cells. For comparison, mock crosses (without *MAT***a** strains) were also performed in parallel to facilitate the discrimination of cytoductants. This procedure selects for the transfer of cytoplasm with *rho*^+^ mitochondria from E^R^ *MAT***a** cells to cells of the *rho*^0^ recipient strain during *kar1-1*-dependent abortive matings [[3](#_ENREF_3)]. The isolated cytoductants were purified and tested for the ability to grow on YPG to confirm their ability to respire, and for the ability to grow on minimal synthetic dextrose medium supplemented uniquely with leucine to exclude the possibility that their colonies contain diploid sectors (diploid cells in the crosses are heterozygous for the *ade2* mutation, and form easily distinguishable big white colonies on the medium used, however, sometimes small diploid sectors, that are not obvious upon visual inspection, occur also in big red colonies). The verified cytoductant clones (respiring isolates with auxotrophic requirements of DFS160) were finally tested for the ability to grow on YPG+erythromycin. The transmission of erythromycin resistance by cytoduction proved the mitochondrial location of the resistance mutation in original E^R^ isolates.

The DFS160 strain was also used in procedures for constructing YAK136 and YAK243. To construct YAK136, first mtDNA from CAB193-1 (Table 6) was transferred by cytoduction into DFS160. The mtDNA from CAB193-1 does not support the respiration in DFS160 cytoductants, but is sufficient for their faster growth and formation of distinctly bigger colonies on the above-described minimal glucose medium in comparison to the *rho*^0^ background (the mitochondrial electron chain is not functional in these strains due to the interrupted *COX3* in mtDNA, but genes coding for subunits of the mitochondrial F_0_ ATPase, which may act in the budding yeast independently from the respiratory chain [[4](#_ENREF_4),[5](#_ENREF_5)], are fully functional). The presence of the correct *rho*^+^ mtDNA in cytoductant DFS160 clones was confirmed by a test cross with the GW22 strain (Table 6). An isolated *kar1-1* strain, carrying *rho*^+^ mtDNA with the *cox3::arg8^m^::(GT)_16_(+2)* reporter gene, was mated with a *rho*^0^ derivative of a *MAT***a** strain obtained after inactivation of the nuclear *ARG8* in FF18733 (FF18733 *arg8*::*URA3*). After the cross, haploid strains with the nucleus of the FF18733 derivative and mtDNA from the carrier *kar1-1* strain was isolated as bigger colonies than *rho*^0^ background on a synthetic minimal (for FF18733 *arg8*::*URA3*) glucose medium. The correct *rho*^+^ FF18733 *arg8*::*URA3* strain was verified by a test cross with the MCC259 strain (Table 6). The same procedure with an intermediary *kar1-1 rho*^+^ mtDNA-carrier strain was followed for the transfer of *rho*^+^ mtDNA from the strain CAB183-1 (mtDNA with the *cox3::arg8^m^::(GT)_16_(+1)* reporter gene) to FF18733 *arg8*::*URA3*, resulting in the isolation of YAK243.

To isolate YAK349, carrying *rho*^+^ mtDNA from originally TF236 (with the *cox3::arg8^m^-1* reporter gene; Table 6), we used a procedure analogous to that described above. Instead of the *kar1-1* *MAT*α strain DFS160, we employed a *rho*^0^ *MAT***a** *kar1-1* JC8/55 as a carrier strain for the transfer of *rho*^+^ mtDNA with the mitochondrial reporter gene from YAK29/1 [[1](#_ENREF_1)], a *kar1-1* *rho*^+^ strain whose mtDNA originates from mating with TF236, into a *rho*^0^ strain derived from PJD1 *arg8*::*URA3.* The presence of the correct mtDNA in the final YAK349 strain was confirmed by a test cross with GW22.

**Characterisation of E^R^ mutations by tetrad analysis**

To isolate heterozygous strains for tracking the E^R^ trait in meiotic progeny, E^R^ isolates lacking the *RAD27* gene (*rad27*Δ::*kanMX4*) were crossed on YPD with a *rho*^0^ derivative of the wild-type strain FF18733 carrying a prototrophic allele of the *HIS7* gene (YAK1070). Diploid cells (His^+^ G418^R^) generated in the crosses were selected by replica-plating on a synthetic complete glucose medium without histidine, but with G418 (at 200 mg/l) and 0.1% glutamine as the nitrogen source instead of the standard ammonium sulfate to allow for the G418 selection on the synthetic medium [[6](#_ENREF_6)]. Diploid clones were purified by streaking for single colonies and checked for the ability to grow on media with glycerol as the sole carbon source. Respiring diploid strains were sporulated according to [[7](#_ENREF_7)]. Tetrads were dissected by micromanipulation on YPD plates and spores from complete tetrads were analyzed for the following markers: histidine prototrophy, resistance to G418 in YPD medium and resistance to erythromycin in YPG medium.

**References**

1. Kaniak A, Dzierzbicki P, Rogowska AT, Malc E, Fikus M, et al. (2009) Msh1p counteracts oxidative lesion-induced instability of mtDNA and stimulates mitochondrial recombination in Saccharomyces cerevisiae. DNA Repair (Amst) 8: 318-329.

2. Styles C (2002) How to set up a yeast laboratory. Methods Enzymol 350: 42-71.

3. Lancashire WE, Mattoon JR (1979) Cytoduction: a tool for mitochondrial genetic studies in yeast. Utilization of the nuclear-fusion mutation kar 1-1 for transfer of drug r and mit genomes in Saccharomyces cerevisiae. Mol Gen Genet 170: 333-344.

4. Giraud MF, Velours J (1997) The absence of the mitochondrial ATP synthase delta subunit promotes a slow growth phenotype of rho- yeast cells by a lack of assembly of the catalytic sector F1. Eur J Biochem 245: 813-818.

5. Kominsky DJ, Brownson MP, Updike DL, Thorsness PE (2002) Genetic and biochemical basis for viability of yeast lacking mitochondrial genomes. Genetics 162: 1595-1604.

6. Tong AH, Evangelista M, Parsons AB, Xu H, Bader GD, et al. (2001) Systematic genetic analysis with ordered arrays of yeast deletion mutants. Science 294: 2364-2368.

7. Amberg DCB, D. J.; Strathern, J. N. (2005) Methods in Yeast Genetics: A Cold Spring Harbor Laboratory Course Manual: Cold Spring Harbor Laboratory Press.
